# Dual Vertically Aligned Electrode‐Inspired High‐Capacity Lithium Batteries

**DOI:** 10.1002/advs.202203321

**Published:** 2022-08-23

**Authors:** Yongbiao Mu, Yuzhu Chen, Buke Wu, Qing Zhang, Meng Lin, Lin Zeng

**Affiliations:** ^1^ Shenzhen Key Laboratory of Advanced Energy Storage Southern University of Science and Technology Shenzhen 518055 China; ^2^ Department of Mechanical and Energy Engineering Southern University of Science and Technology Shenzhen 518055 China; ^3^ Southern University of Science and Technology Shenzhen 518055 China

**Keywords:** dual vertically aligned architectures, lithium metal batteries (LMBs), ultrahigh currents and capacities, vertical graphene nanowalls

## Abstract

Lithium (Li) dendrite formation and poor Li^+^ transport kinetics under high‐charging current densities and capacities inhibit the capabilities of Li metal batteries (LMBs). This study proposes a 3D conductive multichannel carbon framework (MCF) with homogeneously distributed vertical graphene nanowalls (VGWs@MCF) as a multifunctional host to efficiently regulate Li deposition and accelerate Li^+^ transport. A novel electrode for both Li|VGWs@MCF anode and LFP|VGWs@MCF (NCM_811_|VGWs@MCF) cathode is designed and fabricated using a dual vertically aligned architecture. This unique hierarchical structure provides ultrafast, continuous, and smooth electron transport channels; furthermore, it furnishes outstanding mechanical strength to support massive Li deposition at ultrahigh rates. As a result, the Li|VGWs@MCF anode exhibits outstanding cycling stability at ultrahigh currents and capacities (1000 h at 10 mA cm^–2^ and 10 mAh cm^–2^, and 1000 h at 30 mA cm^–2^ and 60 mAh cm^–2^). Moreover, full cells made of such 3D anodes and freestanding LFP|VGWs@MCF (NCM_811_|VGWs@MCF) cathodes with conspicuous mass loading (45 mg cm^–2^ for LFP and 35 mg cm^–2^ for NCM_811_) demonstrate excellent areal capacities (6.98 mAh cm^–2^ for LFP and 5.6 mAh cm^–2^ for NCM_811_). This strategy proposes a promising direction for the development of high‐energy‐density practical Li batteries that combine safety, performance, and sustainability.

## Graphical abstract

Homogeneously distributed vertical graphene nanowalls with abundant exposed edges, as a highly conductive coating layer, are successfully fabricated on 3D multichannel carbon framework (VGWs@MCF), which can effectively regulate Li deposition and accelerate Li^+^ ion transport. The dual vertically aligned architecture electrodes using VGWs@MCF offers significant boost in both ultrahigh rates and capacities (40 mA cm^–2^ and 40 mAh cm^–2^) for LMBs.

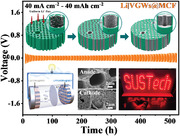

## 1 Introduction

The ever‐growing demand for high‐energy‐density and safe energy storage systems requires the development of battery techniques that provide high specific capacity, stable cycling performance, and a long lifespan.^[^ ^1^, ^2^, ^3^ ^]^ Superior current densities of over 30 mA cm^–2^ (e.g., over 3C rate, full charge within 20 min for a 500 Wh kg^–1^ cell, the goal of the United States Department of Energy) are required to obtain high‐power outputs and realize fast charging of advanced battery systems.^[^ ^4^, ^5^ ^]^ Lithium metal anodes (LMAs) are considered to be a promising anode material for next‐generation rechargeable batteries because of their ultrahigh theoretical specific capacity (3860 mAh g^–1^), extremely low electrochemical potential (−3.040 V vs standard hydrogen electrode), and low gravimetric density (0.534 g cm^–3^).^[^ ^6^, ^7^, ^8^, ^9^ ^]^ Since the 1960s, tremendous attention has been given to LMAs. However, their practical utilization is hindered by several intrinsic defects, including dendritic formation during deposition, instability of the Li metal interface, and infinite volume fluctuation during repeated cycles, causing a fracture in the solid electrolyte interphase (SEI) film, leading to the formation of dendrites and “dead” Li.^[^ ^10^, ^11^, ^12^, ^13^ ^]^ This uncontrollable and dendrite‐forming growth of Li metal can lead to battery short circuits, low cycling coulombic efficiencies, poor cycle lifespans, and safety hazards, which have significantly impeded the application of LMAs.^[^ ^14^, ^15^, ^16^ ^]^

For decades, several strategies have been proposed to solve the notorious issues of LMAs, including the following four approaches. First, inducing additives in the electrolyte is instrumental in stabilizing the SEI films,^[^ ^17^, ^18^ ^]^ which prevents fresh Li metal from further reacting with the electrolyte. Second, prefabricated artificial SEI layer possesses good chemical stability and mechanical properties, benefitting the adaption of the large volume change of the Li metal during the plating and stripping process.^[^ ^19^, ^20^, ^21^ ^]^ Third, employing solid inorganic or polymer electrolytes can partly relieve several safety issues, such as leakage, poor chemical stability, and flammability for liquid electrolytes.^[^ ^22^, ^23^, ^24^ ^]^ Finally, constructing three‐dimensional (3D) conductive scaffolds as the host matrix can not only regulate Li plating and accommodate volume changes but also decrease the local current density due to the enlarged surface area.^[^ ^25^, ^26^, ^27^ ^]^ These strategies have contributed to improving the performance of LMAs; however, the problems of dendrite growth and poor cycling stability remain major concerns in the case of high current density and high areal capacity operation of LMAs.

The design and fabrication of 3D conductive structures as a host matrix for LMAs play a critical role in reducing the local current density and promoting uniform Li plating/stripping owing to an increase in the electroactive surface area compared with interface engineering design, including electrolyte additives, artificial SEI, protective layers, and separator modification.^[^ ^28^, ^29^, ^30^, ^31^ ^]^ Meanwhile, 3D frameworks can effectively support Li and accommodate the stress and strain caused by the large volumetric expansion that occurs during repeated Li plating/stripping cycles, suppressing the formation and growth of Li dendrite. Among the various 3D architecture materials, some typical micro‐/nanostructured frameworks, including metal‐based,^[^ ^32^ ^]^ carbon‐based,^[^ ^33^ ^]^ and metal/carbon hybrid frameworks,^[^ ^34^ ^]^ have been demonstrated to be effective in eliminating the problems mentioned above. Metals, such as stainless steel,^[^ ^35^ ^]^ Cu,^[^ ^36^ ^]^ Ni,^[^ ^37^ ^]^ and Ti,^[^ ^38^ ^]^ have been widely used to develop 3D‐nanostructured Li hosts. Researchers have developed various nanostructures on the Cu‐metal‐based Li hosts such as porous Cu,^[^ ^39^ ^]^ copper nanowires (Cu NWs),^[^ ^40^ ^]^ 3D pie‐like structures,^[^ ^41^ ^]^ vertically aligned Cu,^[^ ^11^ ^]^ and Cu foams.^[^ ^42^ ^]^ Some 3D porous Cu‐Sn, Cu‐Zn, and Cu‐Al alloys have also been used in LMAs.^[^ ^39^, ^43^ ^]^ However, the corrosion of metal‐based 3D hosts is difficult to avoid, and their performance under high current density and high capacity is not optimistic. Furthermore, the 3D conductive carbon architecture has been considered an ideal Li host owing to its light weight, flexibility, high mechanical and chemical stability, high electronic conductivity, low cost, and natural abundance. Various porous carbon host matrices, such as carbon nanotubes (CNT),^[^ ^44^ ^]^ carbon nanofibers (CNFs),^[^ ^45^ ^]^ hollow carbon nanospheres,^[^ ^46^ ^]^ graphite carbon foams,^[^ ^47^ ^]^ multilayer graphene, graphene‐CNT hybrids, reduced graphene oxide, biomass porous carbon,^[^ ^48^ ^]^ and metal‐organic frameworks,^[^ ^49^ ^]^ have been employed to regulate the electron and ion distributions, realize a uniform Li plating and stripping morphology and accommodate volumetric Li expansion without any mechanical destruction. Additionally, other metal/carbon hybrid frameworks, such as CNTs, graphene, and metal‐organic frameworks (MOFs) deposited on various 3D metal frameworks allow the bottom‐to‐top deposition of Li and stabilize the SEI. These novel architectures have been developed to improve the performance of LMAs; however, less attention has been paid to Li metal in the case of high current density and high Li storage capacity.

Some strategies, such as electrochemical deposition, molten Li infusion, and mechanical pressurization, have received increasing attention for achieving high‐energy and power density LMBs to host Li metal into 3D structures. However, there is a large nucleation barrier for the Li deposition on different 3D host matrices due to its “lithiophobic” nature.^[^ ^50^ ^]^ Meanwhile, a higher Li‐ion flux at the host surface, resulting in high Li‐ion diffusion resistance in the bulk electrolyte among the 3D hosts, weakens the effect of the 3D hosts, leading to an overlying deposit of Li and Li dendrites.^[^ ^51^ ^]^ Hence, various surface modifications are proved to be highly effective in reducing Li nuclear energy and guiding Li deposition on the modified 3D matrix, such as those with active coating materials (Si, ZnO, CoN*_x_*, N‐containing functional groups, CuO, MgO, and heteroatom‐doped graphene) and suitable seeding sites (Sn, Ag, and Au). Although significant efforts have been made to introduce lithiophilic materials into various 3D hosts, induce uniform Li nuclei, and alleviate dendrite formation, several issues remain to be addressed. First, in terms of the synthesis process, most 3D conductive hosts are complex and costly, and the inhomogeneous, complicated, and uneconomical preparation technology for the design of lithiophilic‐coating layers limit their large‐scale applications. Second, conventional 3D hosts with relatively large pore sizes (>10 µm) cannot efficiently dissipate large current densities due to their limited surface areas, deteriorating the high‐rate performance of LMAs. Third, the performance of LMAs with high current density and high areal capacity has long been neglected; analysis of this performance would be truly beneficial to the commercialization process. Therefore, it is essential to choose a simple, facile, and economical preparation technology for designing a 3D conductive host with functional surface modification for high current density/areal capacity LMAs.

This study proposes a dual vertically aligned electrode configuration with high conductivity and mechanical stability to develop a dendrite‐free LMA and high‐capacity cathode. In this design, 3D hierarchical ZnO, Co_3_O_4_‐nanoparticle‐anchored vertical graphene nanowalls with abundant oxygen and nitrogen doping were grown on multichannel carbon framework (VGWs@MCF) from carbonized wood as a highly mechanical Li host for dendrite‐free LMAs. Vertically aligned LiFePO_4_ (LFP) and LiNi_0.8_Co_0.1_Mn_0.1_O_2_ (NCM_811_) were fabricated using a facile vacuum filtration method to facilitate Li^+^ transport and reduce Li^+^ diffusion resistance to minimize the Li^+^ transport tortuosity in both the anode and cathode, enhancing the high areal capacities and rate performance. Both fast‐charging Li|VGWs@MCF symmetric cells and LFP|VGWs@MCF (NCM_811_|VGWs@MCF) full cells were fabricated based on the dual vertically aligned electrode structure. Particularly, a 3D conductive MCF with homogeneously distributed vertical graphene nanowalls (VGWs@MCF) host offers the following advantages: (i) 3D VGWs with well‐ordered structure and edge‐enriched surface growth on the carbonized wood matrix via a chemical vapor deposition (CVD) technique significantly regulate the local current distribution and maximize Li storage due to their enlarged specific surface area and porous structure. (ii) The uniform distribution of ZnO and Co_3_O_4_ nanoparticles and N, O, dual‐doped graphene nanowalls endow the VGWs@MCF host with excellent lithiophilicity, guaranteeing uniform Li deposition behavior with a small overpotential. (iii) The VGWs@MCF host possesses high electric conductivity, mechanical strength, and abundant microchannels, which are beneficial for suppressing Li dendrite growth and reducing the risk of short‐circuiting under high current densities and capacities. Additionally, the vertically aligned LFP (NCM_811_) electrode enabled by simply infiltrating commercial cathode materials into the channels obtained ultrahigh mass loading of electroactive materials and low‐tortuosity pathways for Li^+^ ion transport, demonstrating a high capacity and outstanding rate performance. Consequently, stable cycling performance of the assembled symmetric cells over 5200 h was achieved at a current density of 1.0 mA cm^–2^ with an areal capacity of 1.0 mAh cm^–2^. Remarkably, excellent cycling stability was maintained over 800 h under ultrahigh current densities and areal capacities of 40 mA cm^–2^ and 40 mAh cm^–2^, respectively, with a low overpotential of ≈100 mV. Moreover, the LFP| Li|VGWs@MCF full cell delivered a high capacity of 6.98 mAh cm^–2^ at 0.1C, which is comparable to those of other reported high‐performance LFP cathodes. The NCM_811_|Li|VGWs@MCF full cell also exhibited an improved cycling life of over 400 cycles at a high rate of 1C with a discharge capacity of 2.61 mAh cm^–2^. Hence, the insights gained from this work will open new opportunities for developing LMAs with high current densities and capacities.

## 2 Results and Discussion

### 2.1 Fabrication and Characterization of Dual Vertically Aligned Electrodes

**Figure 1:**
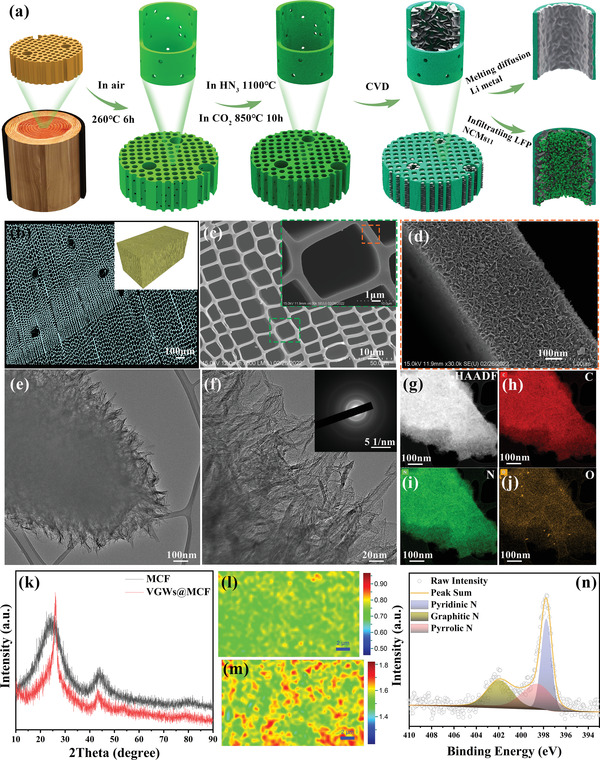
a) Schematic illustration of the design concept of dual vertically aligned electrodes using a 3D VGWs@MCF host; b) A typical micro‐CT image of VGWs@MCF (Inset is 3D panorama); c,d) SEM images, inset in (c) is single channel; e,f) TEM images and g–j) C, N, O elemental mapping images of the VGWs@MCF (Inset in (f) is SAED). k) XRD patterns and l,m) Raman mapping of the intensity ratio of *I*_D_/*I*_G_ and *I*_2D_/*I*_G_ of VGWs@MCF; n) The high‐resolution N 1s patterns of VGWs@MCF.

A schematic illustration of the preparation route for a freestanding dual vertically aligned electrode is shown in **Figure** **1**a. First, natural wood blocks, used as a precursor of the MCF, were cut into pieces of a certain size (Figure S1a,b, Supporting Information), and then the wood slice was heated to 260 °C for 6 h under ambient air to remove moisture. Subsequently, carbonization was performed in a furnace at 1100 °C for 6 h under an ammonia (NH_3_) and carbon dioxide (CO_2_) mixture (Figure S1c,d, Supporting Information). The obvious shrinking of the wood skeleton can be ascribed to the large weight loss (Figure S2, Supporting Information), and only 19.3% of the original weight remained (Figure S3, Supporting Information), demonstrating the formation of a highly lightweight, low‐tortuosity MCF. The role of the porous structure has a great influence on metal deposition. Scanning electron microscopy (SEM) images indicated MCF features with high porosity and low tortuosity with the diameter of 15–20 µm from the top and cross‐section views (Figures S4a–d and S5a, Supporting Information). Furthermore, the carbonization conditions such as temperature (from 800 to 1200 °C) and time (from 3 to 10 h) were changed to explore the pore size distribution, but it had negligible effect on the pore size distribution of MCF (Figure S5b, Supporting Information). Importantly, 3D VGWs grown on MCF (VGWs@MCF) can be readily fabricated using a facile CVD technique. A catalyst‐free approach was employed, and CH_4_ gas was used as the carbon source. The micro‐CT technology presented numerous vertically oriented open microchannels throughout the VGWs@MCF matrix (Figure 1b and Figure S6a–c, Supporting Information). The SEM images also showed a typical surface morphology of the VGWs‐coated carbonized wood (Figure 1c,d), revealing an interconnected VGWs network uniformly covering the carbonized wood substrate. The unique structure of the VGWs was precisely controlled by adjusting the flow rates of hydrogen (H_2_) and CH_4_. When the concentration of CH_4_ was significantly high, the graphene nanowalls became granular, whereas the thickness of the graphene nanowalls increased when the concentration of hydrogen was low (Figure S7, Supporting Information). Transmission electron microscopy (TEM) images demonstrated that VGWs were successfully grown on the MCF (Figure S8, Supporting Information) and possessed abundantly exposed edges (Figure 1e,f). The selected area electron diffraction (SAED) pattern measured from VGWs@MCF comprised of four concentric rings, corresponding to the (002), (100), (102), and (110) planes of the graphitic structure (Inset of Figure 1f), respectively. The height of the VGWs can be modulated by varying the growth time between 3 and 30 h (Figure S9, Supporting Information). For example, the height of the VGWs was ≈200 nm at the growth time of 8 h. High‐resolution TEM (HRTEM) images showed VGWs to have a thin, flexible structure with a sharp triangular top and a large number of small layers of graphene (<ten layers) (Figure S10a–c, Supporting Information). A interlayer distance of 0.352 nm was observed in a single VGW band, which is indicative of the successful growth of graphene via CVD compared with the amorphous carbon structure of MCF (Figures S10d and S11, Supporting Information). Energy‐dispersive X‐ray spectroscopy (EDS) demonstrated a uniform element distribution on VGWs@MCF and MCF, indicating the uniform doping of N and O by NH_3_ and O_2_ plasma treatment (Figure 1g–j). Some trace elements such as Ca and Mg were found in the original MCF because of their absorption from the soil (Figure S12, Supporting Information). The crystalline structure of VGWs@MCF was determined using X‐ray diffraction (XRD) (Figure 1k). The XRD pattern of CW showed a broad peak centered at 22.5°, corresponding to the (002) reflection of the graphitic structure with a d‐spacing of ≈0.395 nm. The (002) peak of the VGWs@MCF upshifted to ≈25°, corresponding to a d‐spacing of ≈0.359 nm, which is consistent with the turbostratic carbon layer observed in HRTEM. The increased intensity and sharp shape of the (002) peak for VGWs@MCF indicate the high crystallinity of the VGWs synthesized via CVD. Furthermore, Raman spectra also confirmed the formation of graphene nanowalls (Figure S13, Supporting Information). Compared with MCF, the G peak (≈1594 cm^–1^), D peak (≈1356 cm^–1^), and 2D peak (≈2727 cm^–1^) were observed in the spectrum of VGWs@MCF, which is a signature of graphene;^[^ ^52^ ^]^ and it can be attributed to the introduction of graphene nanowalls. The 2D peak was absent in original carbonized wood. The presence of high‐intensity D′ (≈2445 cm^–1^) and D′′ (≈2939 cm^–1^) peaks indicates the formation of many sharp edges in the VGWs. The intensity ratio of *I*_2D_/*I*_G_ was 0.61, revealing the property of the graphene nanowalls with few layers. The intensity ratio (≈3.26) of *I*_D_/*I*_G_ for the MCF was higher than that of the VGWs@MCF (≈1.24), which is primarily related to the defects caused by NH_3_ etching within the MCF. Raman mapping (Figure 1l,m) further disclosed the uniformity of VGWs coatings in the MCF based on the *I*_2D_/*I*_G_, consistent with the SEM and TEM observations.

Chemical doping plays a vital role in regulating the lithiophilic properties of graphene for loading Li into the 3D graphene host.^[^ ^53^ ^]^ Therefore, we performed NH_3_ and O_2_ plasma treatments on VGWs@MCF by the plasma‐enhanced CVD method for 10 min to achieve dual‐doped graphene nanowalls of nitrogen and oxygen, resulting in abundant‐exposed lithiophilic edges. X‐ray photoelectron spectroscopy (XPS) analysis was performed to investigate the chemical state properties of VGWs@MCF. The full spectrum of VGWs@MCF proves the existence of C, N, and O elements (Figure S14a, Supporting Information). In the high‐resolution C 1s spectra, the two peaks centered at 284.4 and 285.5 eV are assigned to C–C and C–O, respectively (Figure S14b, Supporting Information). The N 1s spectrum consisted of pyridinic N (398.1 eV), pyrrolic N (399.1 eV), and graphitic N (401.9 eV) (Figure 1n). Normally, pyridinic N/pyrrolic N tends to provide electrons, which can effectively boost the electrical conductivity of the carbon material and offer more lithiophilic sites. The deconvoluted peaks in the O 1s spectrum can be divided into three characteristic peaks: C = O (531.9 eV), C–OH/C–O–C (533.3 eV), and chemisorbed O (534.3 eV). Figure 1a describes that a facile molten‐infusion method was used to infuse Li metal into the VGWs@MCF host, which is of great significance for fabricating large‐scale Li|VGWs@MCF anodes for practical production (Figure S15a, Supporting Information). Specifically, we prepared a lipophilic layer of ZnO and Co_3_O_4_ using a low‐cost solution‐process strategy and sufficient oxidation at 500 °C. As expected, SEM images of the top side and cross‐section showed that ZnO and Co_3_O_4_ nanoparticle coatings dispersed uniformly (Figure S16a–f, Supporting Information), XPS analysis further proved the existence and distribution of ZnO and Co_3_O_4_ in the VGWs@MCF (Figure S17, Supporting Information).^[^ ^54^ ^]^ Subsequently, molten Li metal rapidly infused into VGWs@MCF in less than 2 s (Figure S15b and Video S1, Supporting Information), demonstrating an excellent lithiophilic surface owing to the doped N/O elements and ZnO/Co_3_O_4_ coatings. Furthermore, a 15 × 10 cm^2^ Li composite anode was prepared, which shows significantly potential for practical applications (Figure S15c, Supporting Information).

### 2.2 Stripping/Plating Behavior of 3D Li/VGWs@MCF Anode

**Figure 2:**
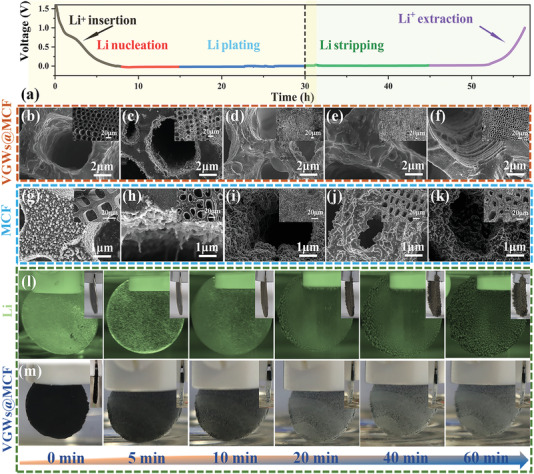
a) Voltage profile of Li plating/stripping process on VGWs@MCF and MCF at 1 mA cm^–2^ with a capacity of 30 mAh cm^–2^. SEM images of VGWs@MCF and MCF morphological evolution: b,g) discharging to 0 V, plating Li of: c,h) 15 mAh cm^–2^ and d,i) 30 mAh cm^–2^; e,j) stripping Li of 15 mAh cm^–2^ and f,k) charging to 1 V. (Inset images are the low‐magnification images). Photographs of Li deposition on VGWs@MCF and MCF at increasing deposition times: from 0 to 60 min for (l) bare Li anode and (m) VGWs@MCF (Inset pictures are the change in thickness of the samples).

The morphological evolution of the Li|VGWs@MCF electrode at different stages of plating/stripping was observed by SEM to clarify the Li deposition behavior in the vertically aligned VGWs@MCF electrode. A current density of 1.0 mA cm^–2^ and a cycling capacity of 30.0 mAh cm^–2^ were employed while considering practical applications. In a benchmark Li‐metal cell, 2D planar copper foil and MCF were used as the current collectors, and a Li metal foil was used as the anode. According to Sand's time model,^[^ ^55^ ^]^ the rough surface of Cu and any microstructured bumps along the current collector could lead to the initial nucleation of Li dendrites and the formation of dead Li during cycling due to the concentrated ion flux (Figure S18, Supporting Information), especially at high current density (Figure S19, Supporting Information). However, the 3D porous construction of VGWs@MCF can significantly decrease the local current density and maximize Li storage owing to its enlarged specific surface area and multichannel structure, thus providing a high‐capacity LMA. A typical voltage profile indicated that the Li plating and stripping process on 3D VGWs@MCF, MCF, and Cu comprised five stages: Li^+^ insertion, Li nucleation, Li plating, Li stripping, and Li^+^ extraction (**Figure** **2**a and Figure S20a, Supporting Information).^[^ ^29^, ^50^, ^56^ ^]^ The overpotential of VGWs@MCF was only 10 mV, which is much lower than that of MCF (23 mV) and bare Cu (55 mV) (Figure S20b, Supporting Information). This suggests a dramatically decreased deposition barrier of Li on the layers of VGWs. In the first stage, the VGWs@MCF host acted as a anode material for lithium‐ion battery, where lithium was intercalated into the N‐doped carbon layer in the form of Li^+^ ions. Figure 2b clearly shows that Li was initially deposited on the microwalls of the VGWs@MCF owing to the lithiophilic surface, resulting in selective nucleation within microchannel walls. In this stage, Li^+^ began to be reduced and nucleate on the lipophilic layer of ZnO and Co_3_O_4_ loaded on VGWs@MCF host. When the Li plating capacity was increased from 1 to 20 mAh cm^–2^, the microchannels were gradually filled (Figure 2c). Notably, little Li was found on the top surface of the VGWs@MCF host. The microchannels were almost completely filled when plating to 30 mAh cm^–2^, (Figure 2d). No microchannels were observed after further increasing to 40 mAh cm^–2^ (Figure S21, Supporting Information). Micro‐CT technology was used to measure the distribution of Li metal in the Li|VGWs@MCF composite anode (Figure S22, Supporting Information). Through the analysis and calculation of the two components in the CT image, we concluded that the volume ratio of Li metal is 44.74%, indicating a high utilization efficiency of VGWs, especially at high areal capacity. We also tracked the change in thickness of the Li|VGWs@MCF anodes at the four stages mentioned above, which showed a negligible change in thickness in the cross‐sectional SEM images (Figure S23a–d, Supporting Information), indicating that Li was completely deposited inside the 3D VGWs@MCF host. In contrast, many irregular Li dendrites grew profusely on the MCF surface after plating at 15 mAh cm^–2^ (Figure 2g,h). Metallic Li was completely deposited on the surface of the MCF, and several dendrites were observed when the capacity varied from 1 to 30 mAh cm^–2^, owing to the lithiophobic nature of MCF. The subsequent process involved Li stripping in the 3D VGWs@MCF, MCF, and Cu. After 15 mAh cm^–2^ Li stripping, the microchannels of VGWs@MCF were re‐exposed (Figure 2e). After the Li metal was completely stripped, the voltage curve from 0.0 to 1.0 V indicated the process of Li^+^ extraction (Figure 2f), the VGWs@MCF remained intact, and VGWs were observed from the top‐view (Figure S24, Supporting Information) and cross‐view (Figure S25, Supporting Information), demonstrating its outstanding reversibility for Li plating and stripping. Nevertheless, a highly irreversible stripping process was observed for bare Cu and MCF. A rough surface and numerous Li pits were observed after stripping (Figure S26, Supporting Information). For the Li|MCF anode, many dendrites and dead Li atoms exist on the surface of the MCF host. Moreover, the Li plating process in the Li foil and VGWs@MCF was monitored in real‐time by operando observation conducted in an electrolytic cell at a high plating current density of 20 mA cm^–2^ for 60 min (Figure S27, Supporting Information). Direct plating of Li onto the Li foil from 0 to 60 min resulted in the continuous formation of many Li dendrites (Figure 2l). The insets show that the thickness of the Li foil increased two to three times compared that of the original Li foil (457 µm) (Figure S28, Supporting Information). In sharp contrast, a slight volume expansion and no obvious dendrites were observed when Li was plated into VGWs@MCF, attributable to the abundant microchannel and uniform layer of VGWs with lipophilic sites (Figure 2m). As a result, VGWs@MCF possessing robust mechanical stability and vertically aligned microchannels with a highly conductive graphene layer exhibited the best capability to suppress the formation of Li dendrites and acquire high‐capacity LMA.

### 2.3 Performance of 3D Li|VGWs@MCF in Symmetrical Cells

**Figure 3:**
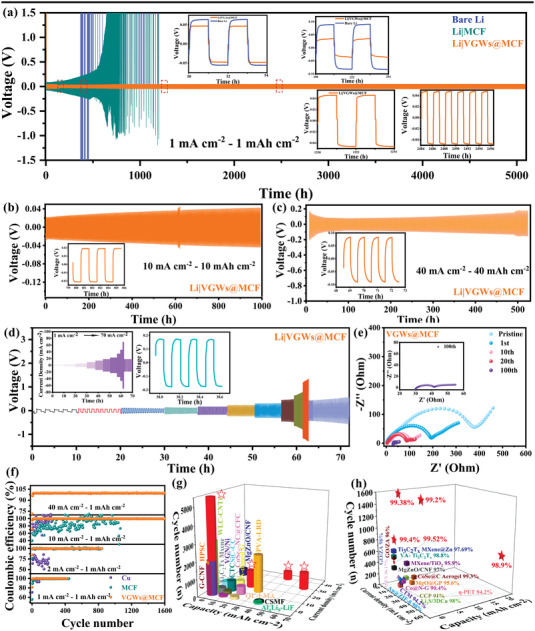
Galvanostatic cycling curves of symmetric batteries of bare Li, MCF, and VGWs@MCF at the current density/capacity of: a) 1 mA cm^–2^/1 mAh cm^–2^; b) 10 mA cm^–2^/10 mAh cm^–2^; c) 40 mA cm^–2^/40 mAh cm^–2^. The insets are the enlarged figures of detailed voltage profiles with different cycle times as indicated. d) Rate performance of symmetrical cells using Li|VGWs@MCF composite anodes measured at current densities of 1–70 mA cm^–2^ at a fixed capacity 1 mAh cm^–2^. The insets are the curves of current density and the detailed voltage profiles at 10 mA cm^–2^/10 mAh cm^–2^. e) Nyquist plots of the initial and cycled Li|VGWs@MCF cell. f) Coulombic efficiencies of the Cu foil, MCF, and VGWs@MCF electrodes with an areal capacity of 1 mAh cm^–2^ at various densities from 1 to 60 mA cm^–2^. g) Comparison of the cycle life of symmetrical cells using Li|VGWs@MCF composite anode and various LMAs under various current densities. h) Comparison of CE of Li|VGWs@MCF anode with various reported LMAs.

The electrochemical cycling performance of the symmetrical Li|VGWs@MCF, Li|MCF, and Li|Li cells was investigated by galvanostatic charge and discharge at various current densities and areal capacities from 1 to 60 mA cm^–2^ and from 1 to 40 mAh cm^–2^, respectively (**Figure** **3**). The Li stripping/plating process was conducted at a fixed capacity of 1 mAh cm^–2^. The cells with the Li|VGWs@MCF anode showed outstanding stable cycle performance at 1 mAh cm^–2^ for up to 5200 h with a lower voltage hysteresis than Li|MCF and bare Li foil (Figure 3a and Figure S29, Supporting Information). Nevertheless, the Li|MCF anode and bulk Li foil show significant polarization even in the early cycles, with hysteresis increasing from 0.4 to 2.0 V. Furthermore, an abrupt voltage drop and severe fluctuation of the voltage profiles occurred after 250 cycles because of internal short circuit (Figure S30, Supporting Information). This is primarily due to the increase in the internal resistance due to inhomogeneous Li deposition and the accumulation of “dead Li” on the bare Li and Li/MCF anodes from the SEM results. The surface morphology of Li|VGW@MCF electrode after different cycles was provided for exploring the stability of the composite structure.^[^ ^57^ ^]^ The Li|VGWs@MCF anode clearly showed that the electrode with vertical channels was filled and covered with a uniform Li metal layer (Figure S31, Supporting Information). Moreover, a super‐flat layer of Li metal was observed on the electrode surface for the Li|VGWs@MCF anode after 200 and 500 h cycles, which can be attributed to its well‐maintained vertical channels structure with abundant vertical graphene nanowalls and enough spaces for storing high capacity Li. Improvements in cycle performance were also observed at high current densities of 10 mA cm^–2^ (Figure S32, Supporting Information), 30 mA cm^–2^ (Figure S33, Supporting Information), and 40 mA cm^–2^ (Figure S34, Supporting Information). The symmetrical cells with the Li|VGWs@MCF anode achieved stable cycling for over 500 h, which is superior to the cells with the Li|MCF and Li|Li anodes. Severe dendrite growth at high current densities and capacities is a critical challenge for the practical application of LMAs. The symmetric Li|VGWs@MCF cells demonstrated stable Li stripping and plating behaviors and an impressive cycling life exceeding 280 and 1000 h when the cycling capacity and current density increased to 2 mAh cm^–2^ and 2 mA cm^–2^ (Figure S35, Supporting Information), and 5 mAh cm^–2^ and 5 mA cm^–2^ (Figure S36, Supporting Information), respectively. Even at a high current density of 10 mA cm^–2^ with an areal capacity of 10 mAh cm^–2^, the cell exhibited long‐term stability for over 1000 h (Figure 3b). Strikingly, the Li|VGWs@MCF symmetric cell showed stable voltage profiles for over 500 h after doubling the current density and capacity up to 40 mA cm^–2^ and 40 mAh cm^–2^, respectively, indicating its superiority as a high‐capacity LMAs (Figure 3c). When aggressive current density and areal capacity of 60 mA cm^–2^ and 30 mAh cm^–2^ were applied, Li|VGWs@MCF indicated a relatively low and exceptionally stable polarization voltage (Figure S37, Supporting Information). The cycling lifespan under various current densities and areal capacities of the Li|VGWs@MCF composite anode in symmetrical cells was compared with relevant results in the literature (Figure 3g and Table S1, Supporting Information). Notably, such excellent long‐term stability under an unparalleled high current density of 60 mA cm^–2^ with an ultrahigh areal capacity of 30 mAh cm^–2^ of our Li|VGWs@MCF anode outperformed other previously reported LMAs.

The rate performances of the bare Li foil, Li|MCF, and Li|VGWs@MCF anodes were further evaluated at 1, 2, 5, 10, 20, 30, 40, 50, 60, and 70 mA cm^–2^ with a varied capacity from 0.5 to 40 mAh cm^−2^. Upon continuous cycling with an increasing current density, the voltage polarization of the Li|VGWs@MCF electrodes increased only marginally at high rates and immediately recovered with their reversal (Figure 3d). On the contrary, the bare Li and Li|MCF electrodes displayed an undesirable, larger overpotential at each rate, particularly at high current densities (Figure S38, Supporting Information). Such low and stable overpotentials indicate the fast ion/electron transport kinetics and robust electrolyte‐anode interface realized at the Li|VGWs@MCF anode, which was further elucidated using electrochemical impedance spectroscopy. Furthermore, Nyquist plots of the symmetrical cells were used to compare the internal resistances of the bare Li, Li|MCF, and Li|VGWs@MCF anodes at different cycles (Figure 3e and Figure S39a,b, Supporting Information). Before cycling, the Li|VGWs@MCF anode possessed a much lower charge transfer resistances (*R*_ct_) of 259.4 Ω than Li|MCF and bare Li. The *R*_ct_ values dropped to 150.4, 189.4, and 164.5 Ω after the first cycle for bare Li, Li|MCF, and Li|VGWs@MCF anodes, respectively. Moreover, the resistances of Li|VGWs@MCF anodes decreased to 68.5 Ω after 20 cycles. However, the resistance of Li foil significantly increased due to the crack of SEI and the formation of dead Li. Both the SEI layer resistance (4.5 Ω) and charge transfer resistance (11.7 Ω) for the Li|VGWs@MCF anodes after 100 cycles of Li plating and stripping were significantly lower than those of the Li foil anode (2208.0 Ω for SEI layer resistance and 660.7 Ω for charge transfer resistance) (Table S2, Supporting Information). The excellent kinetic process and interface stability facilitated the uniform deposition of Li, even at a high rate, eventually eliminating the Li‐metal dendritic issue. The uniform nucleation and deposition of Li on 3D VGWs@MCF improved the Coulombic efficiency (CE) of Li anodes. The Li|VGWs@MCF and Li|MCF showed a stable CE of over 99.5% after 400 cycles with a current density of 1.0 mA cm^–2^ and an areal capacity of 1.0 mA h cm^–2^ (Figure 3f). In comparison, the Cu foil exhibited a rapidly decreasing CE. After varying the current density (60 mA cm^–2^) and deposition capacity (20 mAh cm^–2^), the high CE (over 98%) of VGWs@MCF demonstrated excellent plating and stripping stability (Figure S40, Supporting Information). Notably, such remarkable cycling performances are superior to those of previously reported LMAs (Figure 3h and Table S3, Supporting Information). That is attributed to the relatively high surface area and porous structure of VGWs@MCF, providing a 3D multifunctional host for Li deposition. Therefore, the generation of dead Li was suppressed, and the CE was enhanced using 3D lithiophilic frameworks.

**Figure 4:**
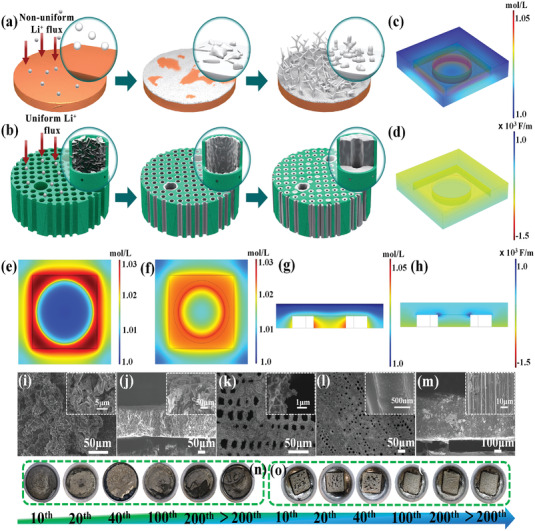
a,b) Schematic diagram of the 2D Cu and 3D VGWs@MCF before and after Li metal deposition. COMSOL Multiphysics simulation of Li^+^ flux distribution in the: e) MCF and c,f,g) VGWs@MCF and d,h) simulation results of the electric field distribution on the VGWs@MCF. SEM images after repeated cycles for i) Li|Cu from top‐side, j) Li|Cu from cross‐section, k) Li|MCF and Li|VGWs@MCF from l) top‐side and m) cross‐section. Optical images showing structural stability of: n) bare Li foil and o) Li|VGWs@MCF anodes after various plating and stripping processes.

The plating behavior of depositing Li on three types of substrates (Cu foil, MCF, and VGWs@MCF) is schematically summarized to clarify the advantages of vertical graphene nanowalls grown on this 3D multichannel carbon framework during the Li plating and stripping process. **Figure** **4**a,b illustrates the mechanisms of Li deposition on the Cu foil and VGWs@MCF, respectively. A large amount of nonuniform Li was deposited owing to the low specific surface area of the planar Cu current collector (Figure S18, Supporting Information); it provided more sites for Li dendrite formation and growth. Consequently, mossy or dendrite‐like Li was distributed on the planar Cu after repeated cycling of plating and stripping (Figure 4i), and Li dendrites grew vertically upward to the Cu substrate, especially at high Li capacity (Figure 4j). However, the 3D hosts of MCF and VGWs@MCF possessed a larger specific surface area than MCF and Cu foil owing to the continuous carbon channels and abundant vertical graphene nanowalls, which provide enough space to store Li and promote the uniform deposition of Li metal in the channels. The highly conductive VGWs layer can significantly release the volume strain, maintaining the integrity of the VGWs@MCF with a stable SEI during the stripping/plating process. COMSOL Multiphysics simulations were also performed to quantitatively reveal the mechanism of the 3D VGWs@MCF host on Li deposition behavior (Figure S41, Supporting Information). The introduction of the VGWs layer was found to effectively regulate the Li^+^ ion and electric field (E‐field) distributions, which is beneficial for Li deposition. The circles in Figure 4c–h represent the microchannels. Evidently, the Li^+^ ions on the upper surface were larger than those within the microchannels of the MCF electrode, resulting in the preferential aggregation of Li on the surface of the MCF skeleton (Figure 4c–e and Figure S42a,b, Supporting Information). These results are supported by the SEM images (Figures 4k and 2g–k). Interestingly, Li metal was first deposited inside the VGWs@MCF, and then the space was gradually filled without any Li dendrites (Figures 2b–f and 4l), which is highly consistent with the simulation results (Figure 4c–f and Figure S42d,e, Supporting Information). The VGWs layer with abundant lithiophilic sites ensured a higher Li^+^ ion distribution inside the microchannels of VGWs@MCF from the cross‐sectional view (Figure 4g and Figure S42f, Supporting Information) compared with the MCF electrode (Figure S42c, Supporting Information), enabling a highly integrated Li metal anode without any thickness change (Figure 4m). The distribution of the E‐field also plays an important role in regulating the deposition behavior of Li. The E‐field distribution on the top of the interconnected MCF was much stronger than that within the channels (Figure S43, Supporting Information), confirming the E‐field polarization in the upper area. However, the E‐field distribution over the entire VGWs@MCF was relatively uniform (Figure 4d,h). This result is favorable for protecting Li metal from dendrite growth during a further deposition process, including the nucleation stage at the inner wall and growth stage in the upward and inward directions of VGWs@MCF. As a result, the deposited Li metal was fully imprisoned in the 3D VGWs@MCF, enabling the long‐term and stable cycling of the as‐formed LMAs. As showed in Figure 4n,o, the Li|VGWs@MCF composite anode maintained high integrity of the electrode structure and robust mechanical strength regardless of the rigorous tests and cycles, whereas the bare Li foil had serious pulverization after short‐term cycles. Therefore, experiments and COMSOL Multiphysics simulations demonstrated that Li|VGWs@MCF offers structural and compositional advantages for developing high‐performance LMAs.

### 2.4 Application of 3D Li|VGWs@MCF Anode in Li‐Ion Battery

**Figure 5:**
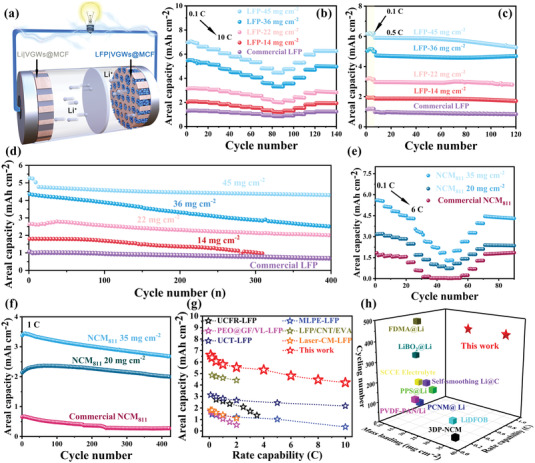
Electrochemical performance of full‐cell with dual vertically aligned electrodes. a) Schematic illustration of LFP|VGWs@MCF (NCM_811_|VGWs@MCF) cathode and Li|VGWs@MCF anode; b) Rate performance from 0.1 to 10 C for LFP|VGW@MCF cathode. The cells cycling performance at: c) 0.5 C and d) 8 C; e) Rate performance from 0.1 to 6 C for NCM_811_|VGWs@MCF cathode and cycling performance at 1 C; Comparison of rate capability and capacity performances of: g) LFP|VGWs@MCF, and h) NCM_811_|VGWs@MCF cathode with various reported cathode in full cells.

Full cells with 3D Li|VGWs@MCF as anode and LFP or LiNi_0.8_Co_0.1_Mn_0.1_O_2_ (NCM_811_) as cathode were assembled to demonstrate the potential use of the Li|VGWs@MCF anode in practical applications. The 3D multichannel frameworks for Li metal inspired us to develop ultrathick 3D vertically aligned electrodes of LFP or NCM_811_ cathodes with a large thickness of 800 µm and ultrahigh active material loading of 45 mg cm^–2^ by infiltrating the commercial cathode materials into the channels of the 3D VGWs@MCF. Furthermore, a vertically aligned LFP or NCM_811_ cathode was introduced into 3D MCFs with abundant VGWs to facilitate Li^+^ transport and reduce Li^+^ diffusion resistance, improving the rate performance and long‐term cycling lifespan. Therefore, we employed a 3D VGWs@MCF structure for both the anode and cathode in the full battery design of dual vertically aligned electrodes (**Figure** **5**a). For comparison, the LFP or NCM_811_ electrodes were fabricated by a conventional blade casting method. Bare Li was chosen as its counterpart, and the assembled cell was labeled as LFP|Li or NCM_811_|Li. The electrochemical performances of the LFP|VGWs@MCF and conventional LFP electrodes were investigated in coin cells. Both the 3D and conventional electrodes showed typical charge and discharge plateaus at ≈3.5 V (Figure S44, Supporting Information). Moreover, the charge–discharge profiles showed that the full cell with the LFP|VGWs@MCF cathode and Li|VGWs@MCF anode had a longer plateau than the conventional LFP electrode. The areal capacity of LFP|VGWs@MCF, especially the ultrahigh capacity of 6.98 mAh cm^–2^ at 0.1 C (1C = 172 mAh g^–1^), increased with increasing LFP mass loading when the mass loading was up to 45 mg cm^–2^, which is consistent with the SEM results (Figure S45, Supporting Information) and the corresponding EDS mapping (Figure S46, Supporting Information). The Brunauer–Emmett–Teller (BET)‐specific surface area of LFP/VGWs@MCF decreases to the 1.1752 m^2^ g^–1^ compared with the VGWs@MCF (1.8449 m^2^ g^–1^), which also proved that the LFP particles were successfully pumped into the graphene channels.^[^ ^58^ ^]^ As shown in Figure 5b, the Li|VGWs@MCF/LFP|VGWs@MCF and Li/LFP full cells possessed commensurate capacities at an initial rate of 0.1 C, indicating 6.9 and 1.2 mAh cm^–2^, respectively. As the rate increased, the full cell of Li|VGWs@MCF/LFP|VGWs@MCF‐45 exhibited high discharge capacities of 6.61, 6.38, 6.06, 5.79, 5.53, 5.32, 4.8, and 4.456 mAh cm^–2^ at 0.1, 0.2, 0.5, 1, 2, 4, 6, and 8 C, respectively. Even at a high rate of 10 C, a high specific capacity of 4.21 mAh cm^–2^ was maintained for the dual vertically‐aligned cell, while the Li/LFP cell only delivered a capacity of 0.54 mAh g^–1^ at 5 C. The cell‐level gravimetric and volumetric energy densities of the LFP/VGWs@MCF electrodes were calculated based on the detailed components of cells (Table S4, Supporting Information). The LFP/VGWs@MCF cathode demonstrated a gradually increasing gravimetric energy density and volumetric energy density with LFP mass loading ranging from 14 to 45 mg cm^–2^. When LFP loading increases to 45 mg cm^–2^, the gravimetric energy density enhanced by 156.61%, compared with the commercial LFP with 9.8 mg cm^–2^ loading (Figure S47a, Supporting Information). Although the volumetric energy density increases with the increase of LFP loading, the overall volumetric energy densities are still low, which caused by the large total volume of the dual vertically oriented electrodes and the low utilization of the 3D VGWs@MCF in cathode materials. The improved rate performance can be attributed to the fast Li^+^ transport in the vertically aligned channels and excellent 3D conductive networks for rapid electron motion, which can be proved by SEM after certain cycling (Figure S48, Supporting Information). It is pretty difficult for the conventional LFP electrode to achieve high loading and good electrical contact, especially when the electrode is cracked and separated from the current collector during the repeated charge–discharge cycle, leading to poor rate performance and cycle lifespan (Figure S49, Supporting Information). The electrical and ionic transportation behaviors of the dual vertically aligned electrodes were further evaluated using four‐point probe conductivity measurements and electrochemical impedance spectroscopy (EIS). The 3D VGWs@MCF was highly conductive with an electronic conductivity of 31.45 S cm^–1^, 2.5 times higher than the original MCF of carbonized wood, demonstrating the superior benefit of the VGWs grown on the MCF as a 3D conductive host. After infiltration with LFP particles, the electrical conductivity of the LFP|VGWs@MCF electrodes decreased from 31.45 to 25.7, 20.2, 17.8, and 15.6 S cm^–1^ for the LFP mass loadings of 14, 22, 36, and 45 mg cm^–2^, respectively. Moreover, the Nyquist plots also demonstrated a smaller charge‐transfer resistance (*R*_ct_, 52 Ω) and electrode–electrolyte interface resistance (*R*_e_, 5 Ω) for the LFP|VGWs@MCF electrode compared with the conventional LFP electrode (*R*_ct_ of 483 Ω and *R*_e_ of 9 Ω) (Figure S50, Supporting Information). After that, we further investigated the cycling performance of LFP|VGWs@MCF electrodes with the mass loading from 14 to 45 mg cm^–2^, delivering high areal capacities of 6.5, 5.6, 4.8, 3.6, and 1.5 mAh cm^–2^, respectively, and high capacity‐retention of 98.5% after 100 cycles for various electrodes (Figure 5c). Moreover, during the long cycling, an initial capacity of 4.5 mAh cm^–2^ with a retention of 90% after 400 cycles (4.3 mAh cm^–2^) was observed for the LFP|VGWs@MCF‐45 electrode (Figure 5d). However, the Li|LFP battery with a mass loading of 10 mg cm^–2^ showed relatively poor cycling stability, with the capacity decreasing to 0.2 mAh cm^–2^ after 200 cycles. The introduction of vertical graphene nanowalls, on the one hand, improved the conductivity of the whole 3D host, which can ensure the rapid conduction of electrons; On the other hand, the vertically distributed nanowalls with rich and exposed edges increased the contact sites and area with the LFP particles, which maintained the conductivity of the whole electrode during the repeated cycles. In addition, the nitrogen doped of vertical graphene nanowalls improved the wettability of the electrode to the electrolyte, thus providing a fast and direct diffusion channel for Li^+^ ions, which enables the whole electrode to realize a high‐rate capability and cycle stability even under high loading (Figure S51, Supporting Information). This work compared the electrochemical performance of different LFP cathodes for mass loading and fast charging to highlight the superiority of VGWs‐modified dual vertically aligned electrodes (Figure 5g and Table S5, Supporting Information). Highly competitive performance of areal capacity and rate capability was obtained in our study, which is attributed to the unique design of dual vertically aligned electrodes with abundant VGWs in the system.

Full cell utilizing Li|VGWs@MCF composite anode paired with NCM_811_|VGWs@MCF was fabricated and compared for its high capacity and rate performance to further verify the remarkable properties and performance of the 3D VGWs@MCF host for dual vertically aligned battery design. The NCM_811_|VGWs@MCF cathode was obtained via a vacuum‐filtration strategy to filter the NCM_811_ cathode material into the VGWs@MCF host (Figure S52, Supporting Information). NCM_811_ particles were evenly distributed in graphene channels and closely contacted with each other, maintaining a highly conductive network structure from the EDS mapping (Figure S53, Supporting Information).The NCM_811_| VGWs@MCF cells exhibited a much better rate capability than the cells assembled with bare Li|NCM_811_(Figure 5e). The NCM_811_|VGWs@MCF electrode delivered high areal capacities from 1.62 to 5.60 mAh cm^–2^ by varying the mass loading of NCM_811_ from 5 to 35 mg cm^–2^. It retained the discharge capacity up to 3.37 and 1.30 mAh cm^–2^ under 2 and 6 C, respectively. Reversible capacity of 0.27 mAh cm^–2^ for 5 mg cm^–2^, 2.02 mAh cm^–2^ for 20 mg cm^–2^, and 2.71 mAh cm^–2^ for 35 mg cm^–2^ were obtained after 400 cycles (Figure 5f), demonstrating fast transport of Li^+^ ion from the vertically aligned channels with low tortuosity. However, the conventional Li|NCM_811_ cells only offered an areal capacity of 0.15 mAh cm^–2^ at 3 C and displayed fast decay during cycles (Figure S54, Supporting Information). The NCM_811_| VGWs@MCF cells demonstrated an enhanced gravimetric energy density of 278.2 Wh kg^–1^, compared with the traditional NCM_811_ electrode of coin‐cell level (120.0 Wh kg^–1^) (Figure S47b and Table S6, Supporting Information). The improved cycling stability of the NCM_811_|VGWs@MCF electrodes should be attributed to the improved structural stability and highly conductive networks of the 3D electrode (Figure S51, Supporting Information). SEM observations of the 3D NCM_811_|VGWs@MCF electrode after 200 charge and discharge cycles further verified that the multichannel architecture and VGWs of the 3D electrode were well maintained, suggesting excellent structural stability (Figure S55, Supporting Information). As shown in Figure S56 (Supporting Information), the red LED can be lightened by the coin full‐cell with high capacity dual vertically aligned electrodes, and can maintain brightness for a long time. This is an extremely competitive performance in terms of high loading and areal capacity for NCM_811_‐based full batteries (Figure 4h and Table S7, Supporting Information).

## 3 Conclusion

A dual vertically aligned electrode using hierarchical 3D MCF hosts with VGWs was designed and fabricated by depositing Li metal and filtrating cathode materials into an optimized 3D conductive network structure. On the anode side, 3D VGWs@MCF possessed high conductivity and sufficient space to promote uniform interfacial Li^+^ migration and effectively prevent perpendicular growth of dendrites. Benefiting from this unique construction, the Li‐based symmetric cell assembled with the Li|VGWs@MCF electrode achieved a long cycle lift of 4500 h at a current density and areal capacity of 1 mA cm^–2^ and 1 mAh cm^–2^, respectively. Furthermore, ultrahigh plating and stripping current densities of 40 and 30 mA cm^–2^, and their capacities of 40 and 60 mAh cm^–2^ were realized, with long cycle lives of 1000 and 1000 h, respectively. The excellent rate and cycling performance of full cells with LFP or NCM_811_ cathodes were also demonstrated. Under a current density of 0.1 C, the areal capacity was promoted to 6.98 mAh cm^–2^ for the LFP (45 mg cm^–2^) electrode, and 5.6 mAh cm^–2^ for the NCM_811_ (35 mg cm^–2^) electrode. Even at a high current of 6 C, areal capacities of 4.8 mAh cm^–2^ for LFP and 1.3 mAh cm^–2^ for NMC_811_ were maintained. These results contribute to the improved Li^+^ transport kinetics by the dual vertically aligned electrode structure in Li|VGWs@MCF and LFP|VGWs@MCF (NCM_811_|VGWs@MCF). This study provides a new strategy for designing high current density and areal capacity electrodes, achieving a series of dendrite‐free metal anodes. Furthermore, it paves the way for energy storage devices with high energy and power densities.

## 4 Experimental Section

Methods and any associated references are available in the online version of the paper.

## Conflict of Interest

The authors declare no conflict of interest.

## Supporting information

Supporting Information

Supporting Information Video 1

## Data Availability

Research data are not shared.
