# Supplementary material for: Dual Vertically Aligned Electrode‐Inspired High‐Capacity Lithium Batteries
Source: Adv Sci (Weinh). 2022 Aug 23;9(30):2203321. doi: 10.1002/advs.202203321 (PMC9596838; doi:10.1002/advs.202203321)
Supplement: Supplementary file 1 — Supporting Information [file ADVS-9-2203321-s001.pdf]

ADVANCED SCIENCE
Open Access

Supporting Information

for *Adv. Sci.*, DOI 10.1002/advs.202203321

Dual Vertically Aligned Electrode-Inspired High-Capacity Lithium Batteries

*Yongbiao Mu, Yuzhu Chen, Buke Wu, Qing Zhang, Meng Lin and Lin Zeng**

Supporting Information for

Dual Vertically Aligned Electrodes-Inspired High-performance Lithium Batteries

Yongbiao Mu,[a, b, c] *Yuzhu Chen,*[a] *Buke Wu,*[a, b, c] *Qing Zhang,*[a, b, c] *Meng Lin*[a] *and Lin Zeng**[a, b, c]

[a] *Shenzhen Key Laboratory of Advanced Energy Storage, Southern University of Science and Technology, Shenzhen 518055, China*

[b] *Department of Mechanical and Energy Engineering, Southern University of Science and Technology, Shenzhen 518055, China*

[c] *SUSTech Energy Institute for Carbon Neutrality, Southern University of Science and Technology, Shenzhen 518055, China*

*Corresponding author. E-mail: zengl3@sustech.edu.cn (L. Zeng)

Experimental Section

Materials

Natural basswood blocks were purchased from Jiangxi Three-Wood Technology Co., Ltd. Cobalt nitrate $Co(NO_3)_2·6H_2O$, Zinc nitrate $Zn(NO_3)_2·6H_2O$, ethanol (C_2H_5OH), N-methyl-2-pyrrolidone (NMP) were purchased from Shanghai Macklin Biochemical Co., Ltd.. Graphite plates were purchased from JiangSu Shenzhou Carbon Co., Ltd. Ar (purity: 99.99%), NH_3 (99.999%), O_2 (99.999%), CH_4 (99.999%) and H_2 (purity: 99.99%) were purchased from Shenzhen Xiangyuan Industrial Gas Co., Ltd.

For coin-cell, the electrolyte was purchased from Guangdong Canrd New Energy Technology Co., Ltd. Conductive carbon black (Super P), Lithium iron phosphate ($LiFePO_4$, LFP), Ni-rich layered oxides $LiNi_{0.8}Co_{0.1}Mn_{0.1}O_2$ (NCM_{811}), Li foil (0.45mm, 99.9%), Cu foil (9 μm, 99.99%), Al foil (20 μm, 99.99%), polyvinylidene fluoride (PVDF) and Celgard 2500 membrane were purchased from MTI Corporation.

Synthesis of Multichannel Carbon Framework

The multichannel carbon framework (MCF) was obtained via stabilization and carbonization processes. In detail, the natural basswood was first cut into the desired size (discs with the diameter of 12, 14 and 16 mm) and thickness (1, 1.5 and 2 mm) along the radial direction. Then, the wood discs fixed by graphite plate were stabilized in the air at 260 ℃ for 6 h. The carbonization process was implemented at 1100 ℃ for 10 h in an ammonia (NH_3, 50 mL min^{-1}) flow. The carbonized discs were activated in a carbon dioxide (CO_2, 100 mL min^{-1}) flow at 750 ℃ for 12 h to obtain an activated multichannel carbon framework.

Synthesis of VGWs@MCF composite

The VGWs@MCF composite was prepared via chemical vapor deposition (CVD) technology for growing vertical graphene nanowalls on the multichannel carbon framework. First, the as-prepared MCF was placed in the furnace center through a corundum crucible container. Second, the furnace was heated to 1100 ℃ at a rate of 5 ℃ min^{-1} under NH_3 (100 mL min^{-1}). Third, the methane (CH_4, 20-60 mL min^{-1}) used as a carbon source was introduced into the quartz furnace when the temperature reached 1100 ℃. The vertical graphene nanowalls (VGWs) can be grown successfully between 1000 ℃ and 1200 ℃ on MCF by introducing H_2 (80-160 mL min^{-1}). In this period, the concentrations of H_2 and CH_4 varied with the temperature. The VGNs with different heights can be obtained by controlling the holding time (3-12 h) at constant temperature. After growth, the furnace was naturally cooled to room temperature. The VGWs@MCF composite was obtained finally.

Synthesis of Li|VGWs@MCF composite anode

The Li|VGWs@MCF composite was prepared through a one-step melt-diffusion process. Before preparing the composite electrode, a plasma bombardment experiment was carried out in NH_3 and O_2 for 10 min each, which enabled the doping of N and O in the rich graphene edge defects. And then, $Co(NO_3)_2·6H_2O$ and $Zn(NO_3)_2·6H_2O$ were dissolved in ethanol with a concentration of 80 mg mL^{-1}. The VGWs@MCF composite was immersed into the above-mixed solution, followed by drying and calcining at 550 ℃ for 30 min in a muffle furnace. Finally, lipophilic zinc oxide (ZnO) and cobalt oxide (Co_3O_4) were successfully coated on the carbon channel wall of VGWs@MCF

composite. During the melt-diffusion process, the lithium block was heated to 300 ℃ in an Ar-filled glove box to make it melt. And then VGWs@MCF composite with lipophilic coating was dipped into molten Li, as shown in Movie S1. The molten Li was rapidly diffused into the multichannel of the VGWs@MCF within 5 seconds, obtaining the Li|VGWs@MCF composite anode. The Li percentage in the Li|VGNs@MCF composite was approximately 50 wt%.

Synthesis of LFP|VGWs@MCF, NCM_{811}|VGWs@MCF composite cathode

The LFP|VGWs@MCF and NCM_{811}|VGWs@MCF composite cathode were prepared through simple vacuum filtration method. First, commercial LFP (NCM_{811}) cathode material (≈ 200 nm) was dispersed in NMP solvent to make the slurry without a conductive agent and binder. Second, the VGWs@MCF composite was placed on a vacuum apparatus and added the slurry drop by drop. The process lasted half an hour to make the cathode slurry infiltrate into the channels of the VGWs@MCF. Third, the samples of as-infiltrated were kept in a vacuum oven at 80 ℃ for 12 h, resulting in the final product of the LFP|VGWs@MCF (NCM_{811}|VGWs@MCF) composite cathode. The mass loadings of LFP (NCM_{811}) can be calculated by direct weighting, which was adjustable through varying the time of infiltrating and controlling the total amount of slurry dropping. In this work, the mass loadings of the cathode (LFP or NCM_{811}) were controlled between 10 and 50 mg cm^{-2}.

Materials Characterizations

The morphologies were characterized by Hitachi SU-8230 field emission scanning electron microscopy (FESEM, Hitachi SU-8230). Transmission electron microscopy (TEM), energy-dispersive X-ray analysis (EDX) and elemental mapping were

performed using a Talos instrument with an acceleration voltage of 300 kV. X-ray diffraction (XRD, Bruker Advance D8, Ultima IV with D/teX Ultra with Cu-Kα radiation) was employed to characterize the crystalline structures of samples with a scanning rate of 5° min^{-1}. X-ray photoelectron spectra (XPS, Escalab 250Xi) were acquired on a Thermo SCIENTIFIC ESCALAB 250Xi with Al Kα (hυ = 1486.8 eV) as the excitation source. Raman spectra were performed on a HORIBA LabRAM HR Evolution using a 532 nm laser as the excitation source. A typical four-probe method (RTS-8) was used to measure the conductivity of the as-prepared VGNs@MCF composite materials. The micro-CT was detected by a Diondo D2 micro-CT scan system.

In Situ Optical Microscope Characterization

The cells for the in situ optical microscope observation were assembled with the electrolytic cell bought from Beijing Instrument Electric Technology Co., Ltd. The working electrodes were the VGWs@MCF composite and Li metal, respectively. The counter electrodes were both Li metal. The electrolyte used was 1M $LiPF_6$ dissolved in ethyl carbonate + dimethyl carbonate + ethyl methyl carbonate (1:1:1 by volume). Neware multichannel battery testing device was used to supply the power, and the current density applied was 20 mA cm^{-2}.

Modeling for distribution of electric field and lithium-ion

1) Governing Equations

3D Nernst-Planck formulation accounting for diffusion and electromigration in the bulk electrolyte was used to study the distribution of electric field and lithium ion for the Li anode with different physical models. The governing equations listed below considering mass balance, species transport, electro-neutrality, and current conservation are given as:

$$\nabla \cdot J_i = R_i \qquad (1)$$

$$J_i = -D_i \nabla c_i - z_i u_i F c_i \nabla \phi_l \qquad (2)$$

$$\sum_i z_i c_i = 0 \qquad (3)$$

$$\nabla \cdot i_l = F \sum_i z_i R_i \qquad (4)$$

where J_i is the mass flux for each species, $R_i = \frac{-v_i i_{loc}}{2F}$ is the electrochemical reaction source term, D_i is the diffusion coefficient ($D_{Li} = D_{PF_6} = 1.22 \times 10^{-6}$ cm^2/s), u_i is the mobility (defined by the Nernst-Einstein equation), c_i is the concentration, z_i is the charge number, $i_l = \sum_m i_{loc,m}$ is the current density of electrolyte (m indicates the charge transfer electrode reaction), ϕ_l is the electrolyte potential, v_i is the stoichiometric coefficient, i_{loc} is the local current density at the electrode surface, and F is the Faraday constant.

2) Geometrical parameters, boundary conditions and numerical solution

The 3D geometrical dimensions, cross-sections, and major boundary conditions of VGWs@MCF and MCF are shown in Figure S37a-37b. The domain of bulk solution was rectangular shape with a dimension of 40 × 40 × 10 μm, and dimension of porous wood anode was 33 × 33× 1 μm. The pore size of the wood anode was 6 μm with 6.5 μm spacing between pores and the thickness of vertical graphene nanowalls (VGWs) was 1 μm. The marked dotted lines were the position for showing the selected cross sections (DO = HO =7.5 μm). Boundary conditions of the two models were shown in Figure S37c-37d. The initial concentration of Li^+ and the magnitude of the external electric current density were set at the top of bulk solution (yellow line). The concentration of Li^+ was set to be 1 M, and the external electric current density flows into Li anodes was set to be 40 mA/cm^2. The surface of Li anodes has a boundary

electric potential of 0 V (red line). The rest boundaries are insulation.

Two models were solved in a commercial software COMSOL Multiphysics using the finite element method. Two models have been ensured the grid-independence by an increasing number of mesh element numbers from 1.0×10^5 to 3.2×10^6. The mesh numbers of computation domains were discretized into 1.4×10^6 and 1.2×10^6 elements for VGWs@MCF and MCF, respectively, to ensure the variation of mean Li concentration and current density at the Li anodes less than 0.01% as a further increase of mesh numbers. A fully coupled Newton method, using a PARDISO solver, with a relative error of 1×10^{-5} was used to solve all coupled PDEs involved.

Electrochemical Performance Assessment

To evaluate the electrochemical behavior and the Coulombic efficiency (CE) of the Li plating and stripping, CR2032-type coin cells were assembled in an Ar-filled glove box (H_2O and O_2 contents <0.01 ppm) with the MCF, VGWs@MCF or planar Cu as the working electrode, Li foil as the counter/reference electrode and a Celgard 2500 separator. The electrolyte used was 1 M $LiPF_6$ dissolved in ethyl carbonate + dimethyl carbonate + ethyl methyl carbonate (1:1:1 by volume). The electrochemical performance was tested using a Neware battery test system (Shenzhen, China) at 30 ℃. To evaluate the CE, 1 mAh cm^{-2} (vary from 0.5 to 40 mAh cm^{-2}) of Li was plated onto the VGWs@MCF or planar Cu and then charged to 1V (vs. Li^+/Li) to strip the Li for each cycle at different current densities (vary from 0.5 to 60 mA cm^{-2}). Before the CE tests, the cells were first activated for 5 cycles with the voltage range of 0-1.0 V at 0.5 mA cm^{-2} to acquire a stable solid electrolyte interphase (SEI) and remove the impurities

on the electrodes.

To evaluate the cycling stability and voltage hysteresis, the symmetric cells were assembled using Li/VGWs@MCF, Li/MCF composite anodes or Li foils and performed at various current densities (0.5-60 mA cm^{-2}) with different capacities of Li deposition (0.5-40 mA cm^{-2}). For comparison, 10 mAh cm^{-2} Li was first predeposited onto the MCF and the planar Cu foil into half cells, forming the Li|MCF anode and the Li|Cu anode, respectively. Then, the Li/MCF and Cu@Li anode were respectively extracted from the half cells, and symmetrical cells were assembled with Li|VGWs@MCF anode, Li|MCF anode (control group), and bare Li (control group), respectively. Galvanostatic cycling was conducted during Li plating/stripping while the potential was recorded over time. Electrochemical impedance spectra (EIS) measurements were carried out using a CHI 760D electrochemical workstation with a frequency range of 100 kHz to 100 mHz (CHI 760D, Shanghai CH Instruments Co., China).

Li|VGWs@MCF Full Cell Assessment

For full cells, the cathode electrodes were obtained by infiltrating commercial cathode powders into the VGWs@MCF composite. The free-standing cathodes were obtained after removing the solvent from the slurry. For comparison, LFP (NCM_{811}), conductive additives (Super P) and polyvinylidene fluoride (PVDF) were added to N-methylpyrrolidone (NMP) with a mass ratio of 8:1:1, and the mixture was stirred for 4 h to obtain a slurry which was then blade-coated onto aluminum foil. For LFP cathode, the cycling curves were measured at 0.1 and 1 C (1 C=170 mA g^{-1}), and rate curves

were tested at 0.2-10 C in the voltage window of 2.5-4.2 V. For NCM_{811} cathode, the cycling curves were measured at 0.1 and 1 C (1 C=190 mA g^{-1}) and rate curves were tested at 0.2-10 C in the voltage window of 3.0 V-4.3 V. In full cells, the capacities were calculated based on the active mass of LFP or NCM_{811}. All the electrochemical measurements were performed at 30 ℃.

The gravimetric energy density ($\boldsymbol{E_g}$) of the full cell is calculated based on the following equations[1]:

$$E_g\ (Wh\ kg^{-1}) = \frac{U\times C_{cell}}{m_{cell}} = \frac{U\times q_{CAM}\times m_{CAM}}{m_{cathode}+m_{anode}+m_{eletrolyte}+m_{others}} \quad (1)$$

where U, C_{cell} represent the average operating voltage of the cell (V) and areal capacity of the cell (mAh cm^{-2}), respectively; q_{CAM} and m_{CAM} describe specific capacity of cathode active material (CAM, mAh g^{-1}), and single-sided CAM areal mass loading (g cm^{-2}), respectively. m_{cell}, $m_{cathode}$, m_{anode}, $m_{electrolyte}$, and m_{others} are areal mass (g cm^{-2}) of the cell, cathode, anode, electrolyte and others (current collector and separator), respectively.

The volumetric energy density ($\boldsymbol{E_v}$) of the full cell is calculated based on the following equations[1]:

$$E_v\ (Wh\ L^{-1}) = \frac{U\times C_{cell}}{v_{cell}} = \frac{U\times q_{CAM}\times m_{CAM}}{v_{cathode}+v_{anode}+v_{others}} \quad (2)$$

where U, C_{cell}, $q_{CAM,}$ m_{cell} represent the average operating voltage of the cell (V), areal capacity of the cell (mAh cm^{-2}), specific capacity of cathode active material (CAM, mAh g^{-1}), and single-sided CAM areal mass loading (g cm^{-2}), respectively. $V_{cathode}$, V_{anode}, and V_{others} are the volume (cm^3) of cathode, anode, and others (current collector and separator), respectively. The volume of electrolyte was ignored due to the pores inside cathode and separator for accommodating electrolyte in the cells.

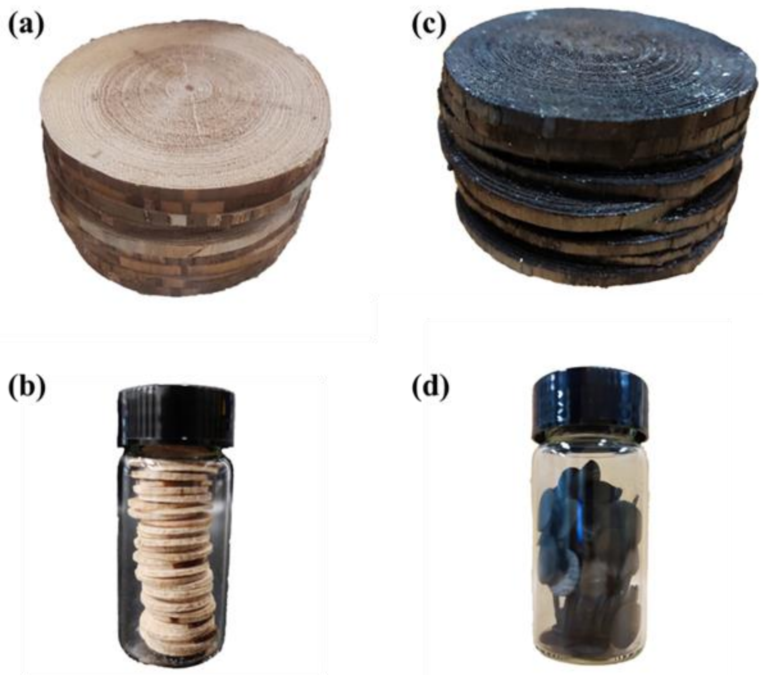

Figure S1. Photographs of (a) the natural wood blocks and (b) after cutting into pieces; (c) the carbonized wood (named MCF) and (d) the MCF pieces.

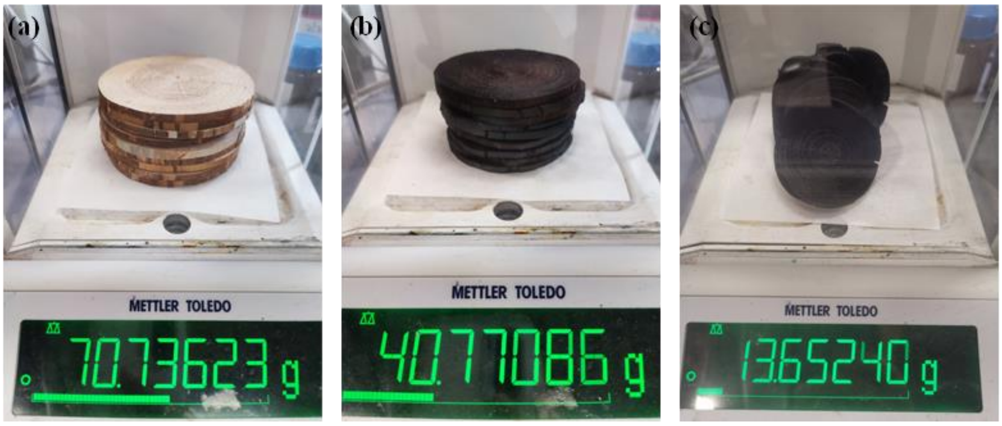

Figure S2. The quality change at different stages of (a) natural wood, (b) stabilization and (c) carbonization process.

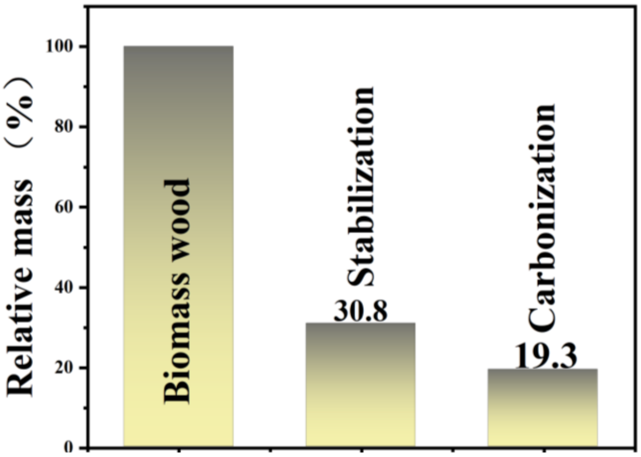

Figure S3. The relative quality change at different stages of (a) natural wood, (b) stabilization and (c) carbonization process.

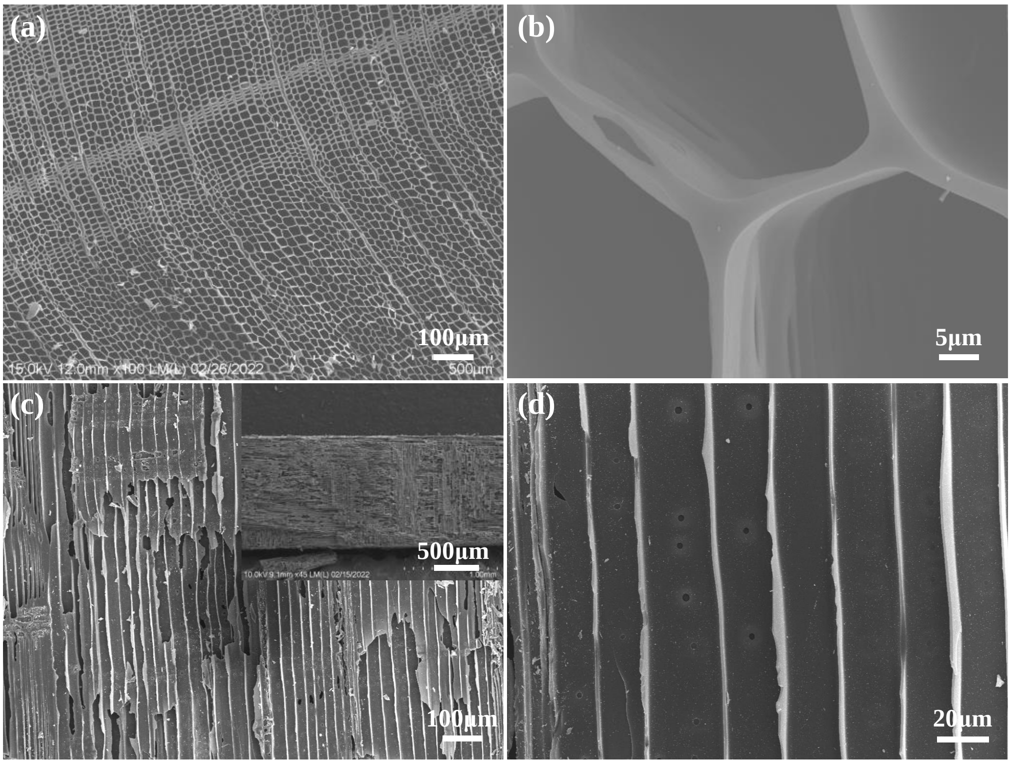

Figure S4. SEM images of MCF from (a, b) top-side and (c, d) cross-section view. The inset is SEM image of MCF electrode from cross-section, showing the thickness of the electrode.

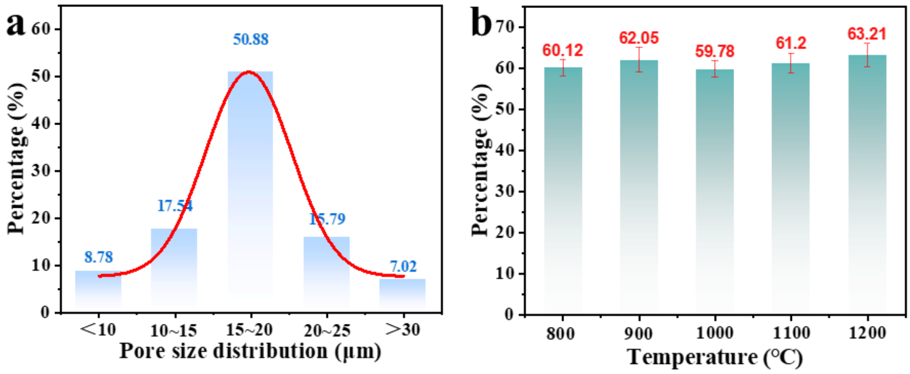

Figure S5. a) Pore size distribution of of MCF, b) percentage of pore size distribution of 15~20 μm at different temperature.

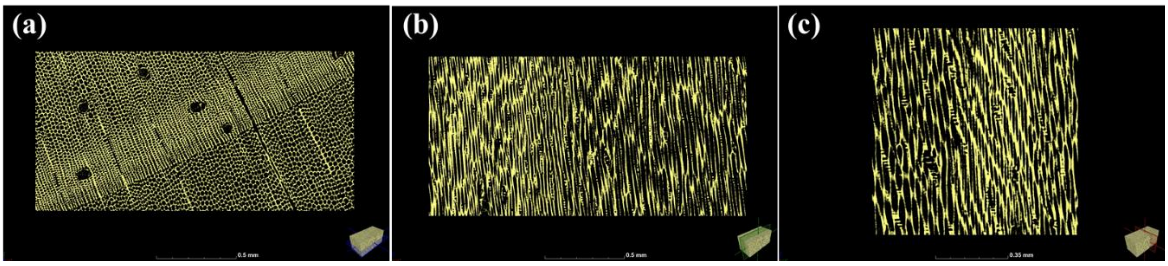

Figure S6. Micro-CT images of VGWs@MCF for (a) top-side image, (b) right-side image and (c) left-side image.

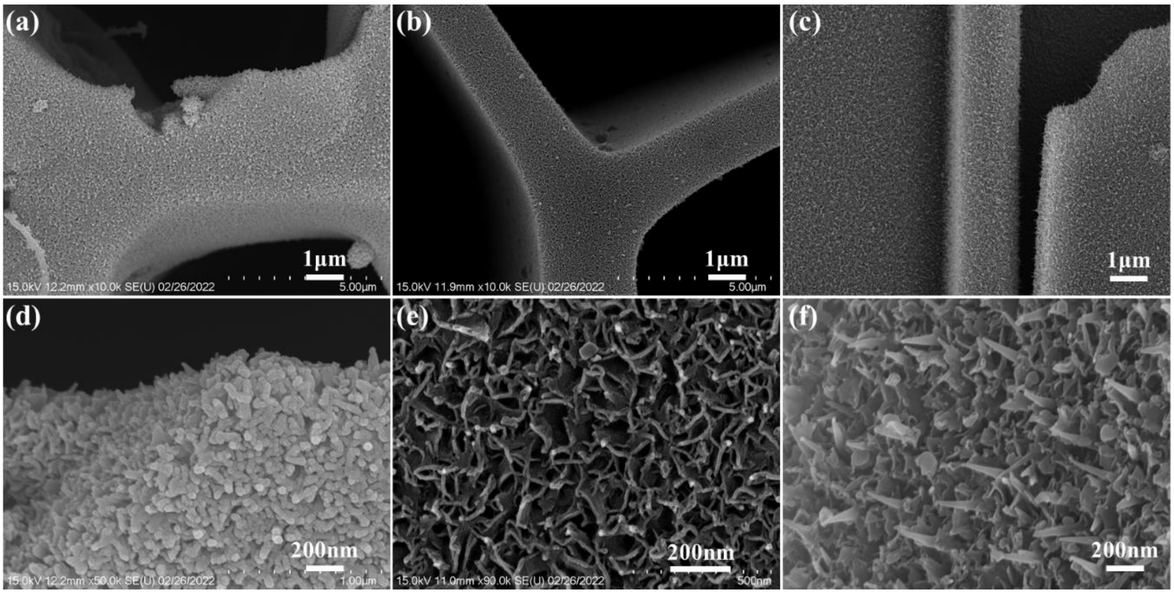

Figure S7. SEM images of VGWs@MCF for (a, d) H_2/CH_4 concentration: 100/40 mL/min, (b, e) H_2/CH_4 concentration: 100/20 mL/min and (c, f) H_2/CH_4 concentration: 120/20 mL/min.

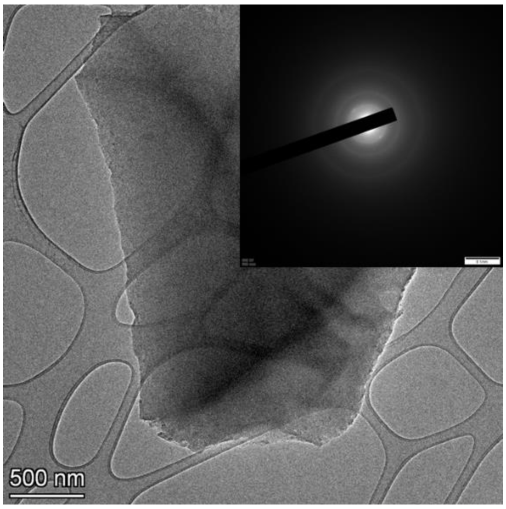

Figure S8. TEM images of MCF (Inset picture is SAED).

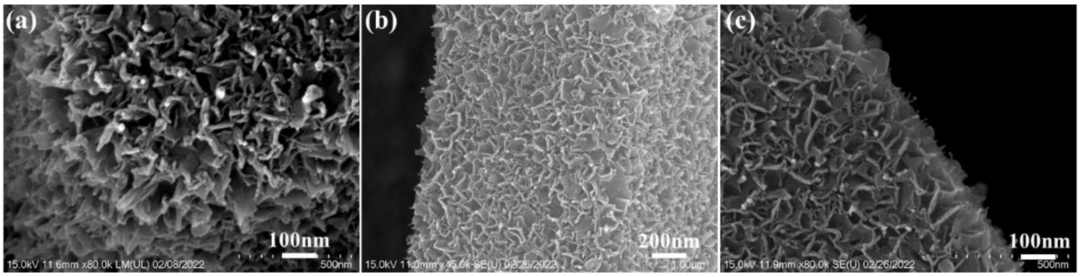

Figure S9. SEM images of VGWs@MCF during different growth time for (a) 30h, (b) 20h and (c) 10h.

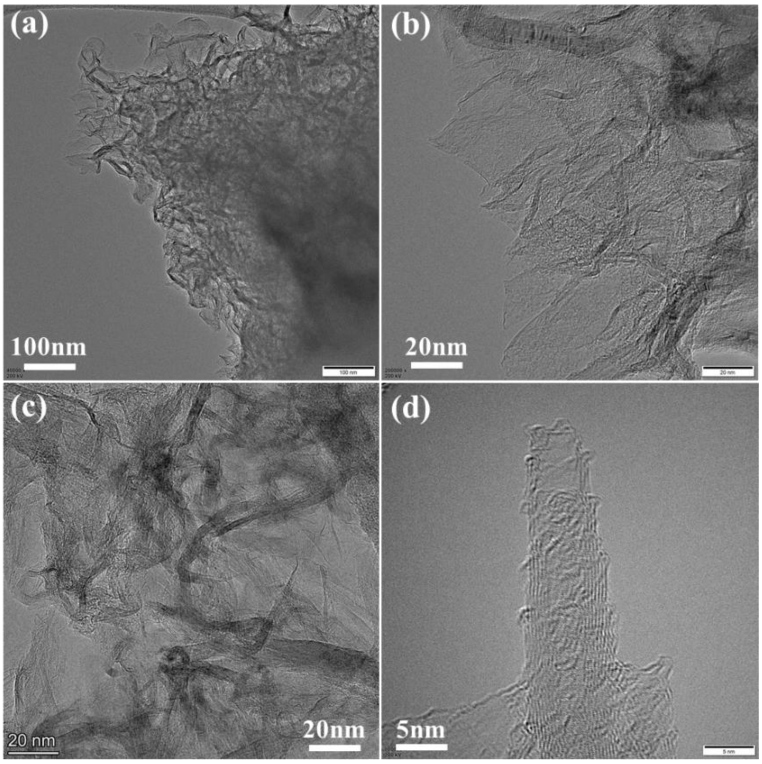

Figure S10. TEM images of VGWs@MCF for (a) abundant vertical graphene nanowalls, (b) sharp triangular top, (c, d) few layers graphene structure.

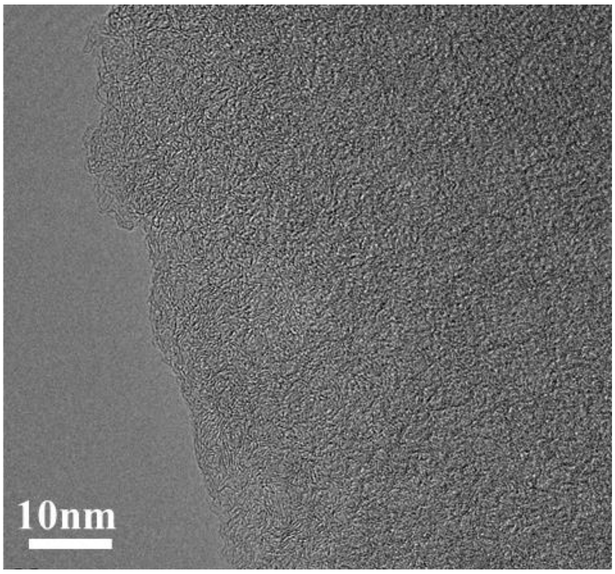

Figure S11. HRTEM images of MCF.

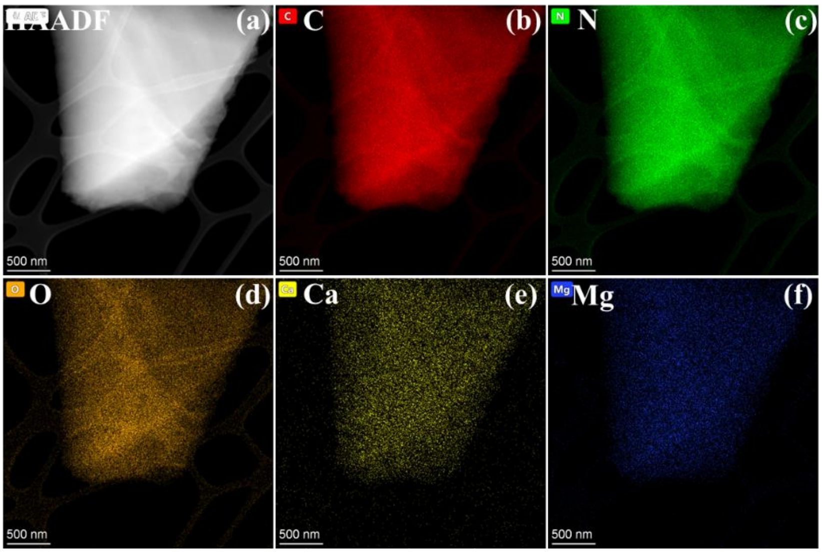

Figure S12. TEM element mapping analysis of MCF for (a) HAADF, (b) C, (c) N, (d) O, (e) Ca and (f) Mg.

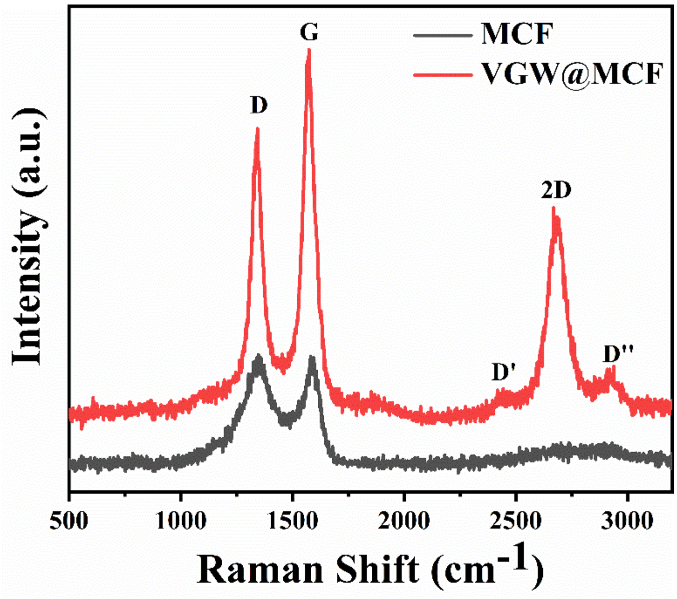

Figure S13. Raman spectra of MCF and VGWs@MCF.

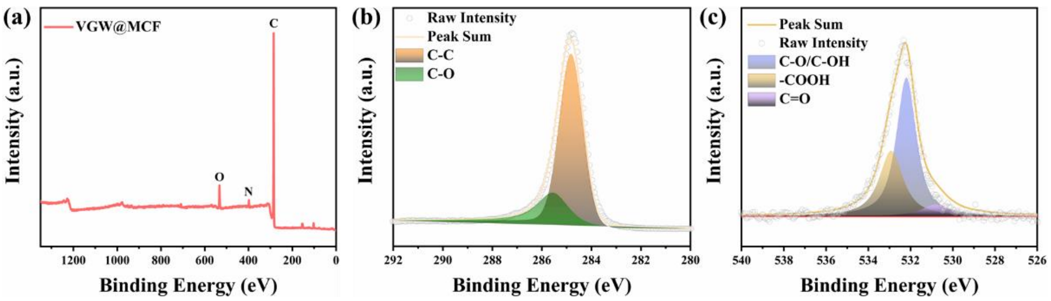

Figure S14. XPS spectra of VGWs@MCF: (a) full spectra, the high-resolution spectra of (b) C1s and (c) O1s.

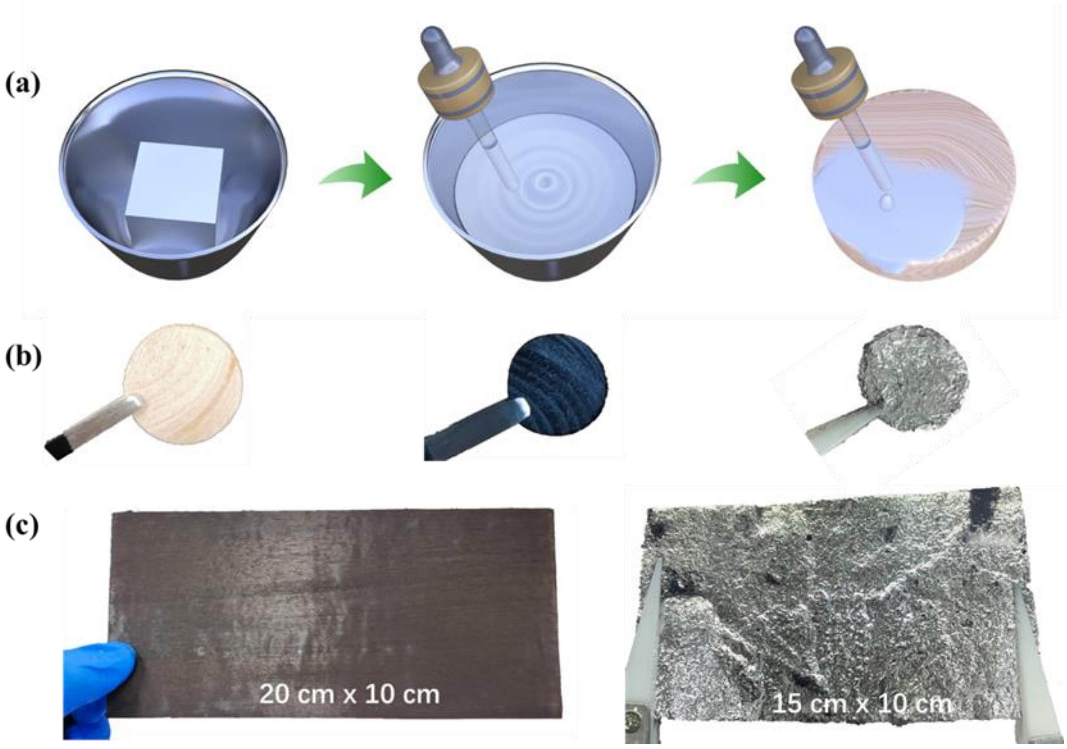

Figure S15. (a) The diagrammatic sketch of the preparation process of Li|VGWs@MCF anode, (b) the optical photos of the obtained electrode and the large-size Li metal composite anode.

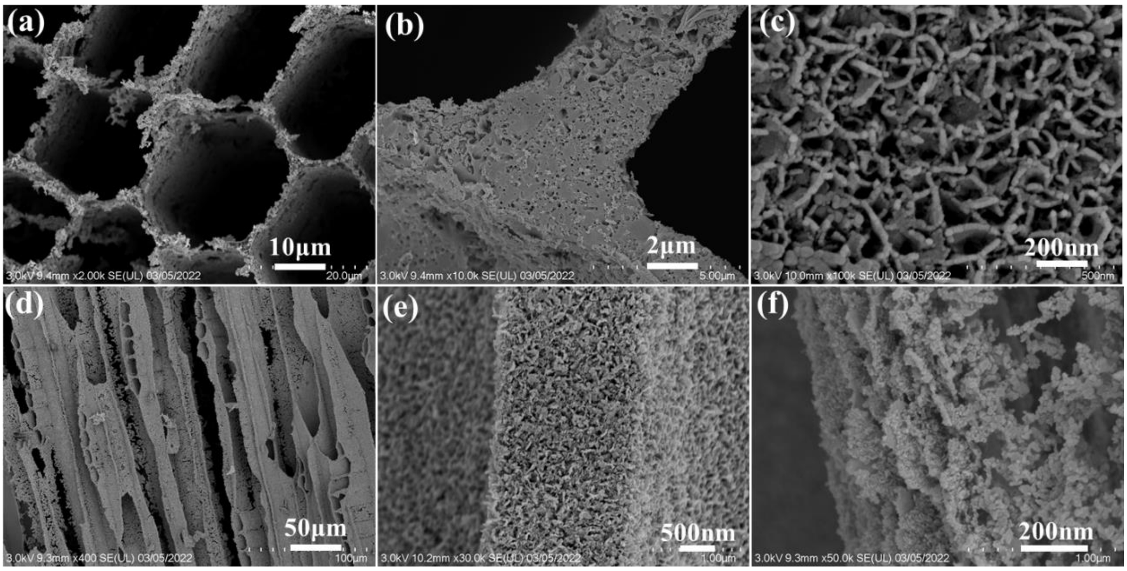

Figure S16. Structural regulation of lithiophilic surface. SEM images of VGWs@MCF after soaking in $Zn(NO_3)_2/Co(NO_3)_2$ solution and oxidation treatment: (a-c) top-side and (d-f) cross-sectional view.

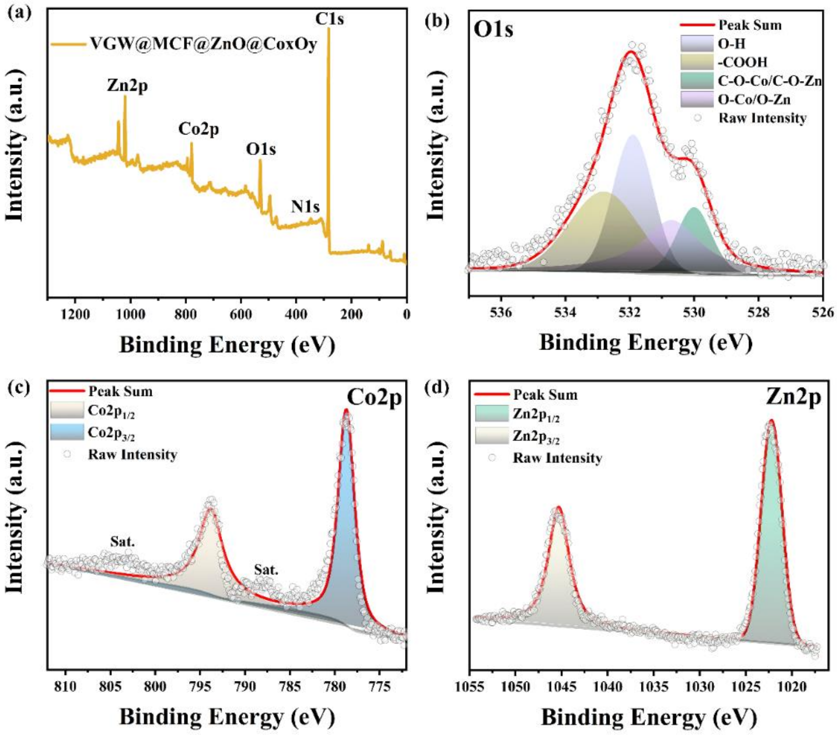

Figure S17. XPS spectra of ZnO and Co_3O_4 coatings on the VGWs@MCF: (a) full spectra, the high-resolution spectra of (b) O1s; (c) Co2p and (d) Zn2p.

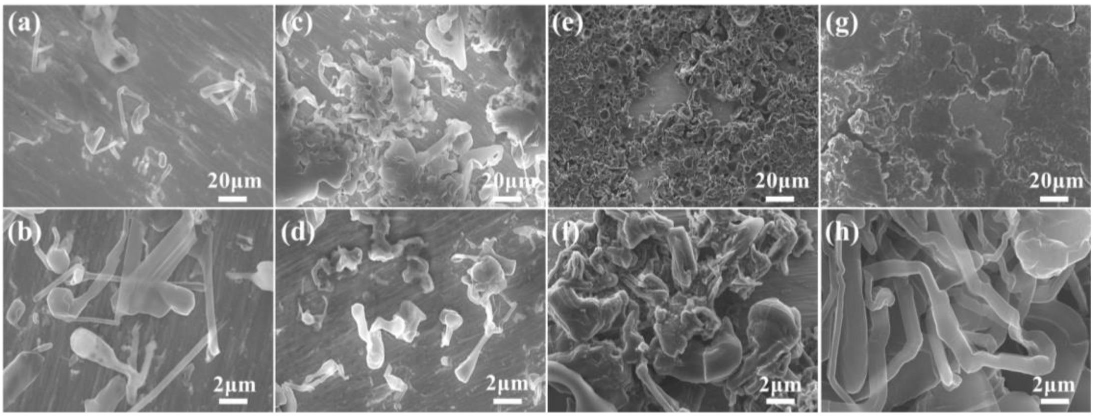

Figure S18. SEM images of Li metal deposited at 1 mA cm^{-2} on 2D planar copper foil from 1 mAh cm^{-2} to 20 mAh cm^{-2}: (a, b) 1 mAh cm^{-2}; (c, d) 5 mAh cm^{-2}; (e, f) 15 mAh cm^{-2} and (g, h) 20 mAh cm^{-2}.

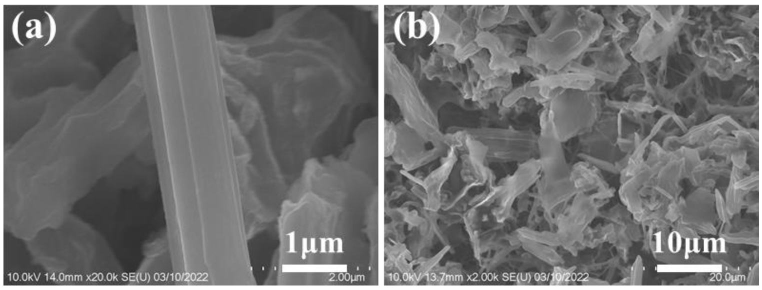

Figure S19. SEM images of Li metal deposited at 10 mA cm^{-2} for a capacity of 10 mAh cm^{-2} on 2D planar copper foil.

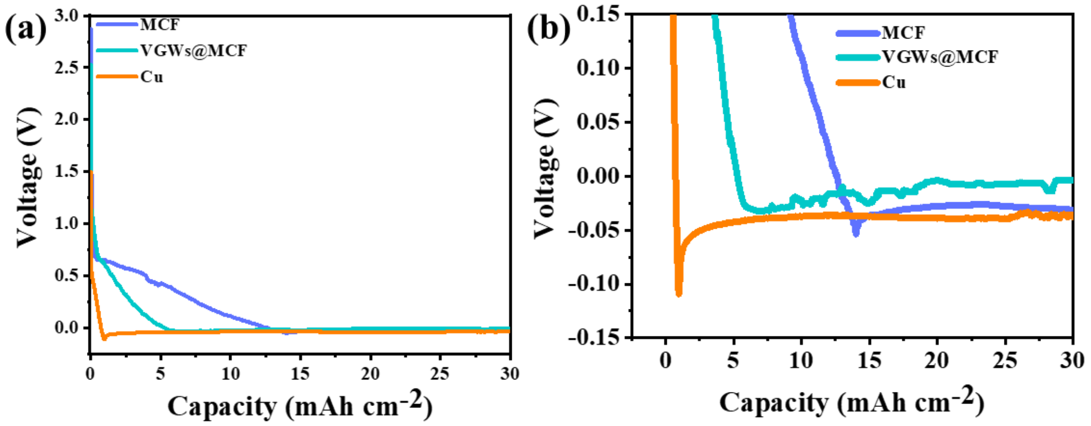

Figure S20. (a) Voltage profiles of Li plating on VGWs@MCF, MCF, and Cu foil. The nucleation voltage of VGWs@MCF is 10 mV, lower than that of MCF (23 mV) and Cu (55 mV).

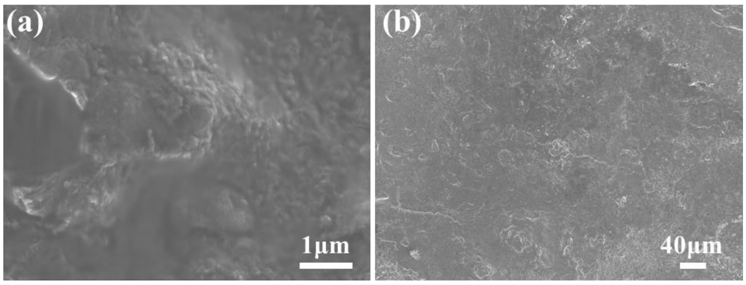

Figure S21. SEM images of Li|VGWs@MCF composite anode when plating to 40 mAh cm^{-2}.

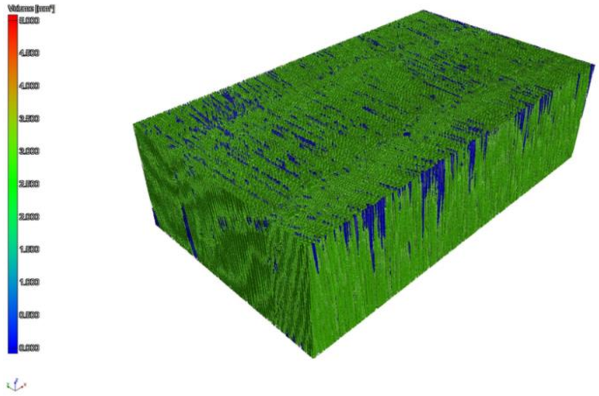

Figure S22. Micro-nano CT image of Li|VGWs@MCF composite anode.(Green represents Li metal and purple represents carbon skeleton).

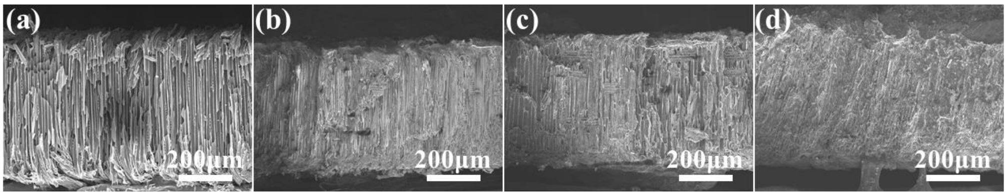

Figure S23. Cross-sectional SEM images of Li|VGWs@MCF composite anode when varying the capacities from a) 1 mAh cm^{-2}, b) 15 mAh cm^{-2} to c) 30 mAh cm^{-2} and d) 40 mAh cm^{-2}.

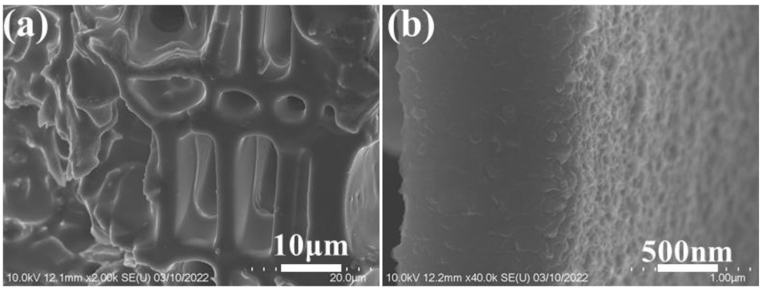

Figure S24. SEM images of Li|VGWs@MCF composite anode when further stripping Li metal beyond 30 mAh cm^{-2} to reveal the structure of vertical graphene nanowalls.

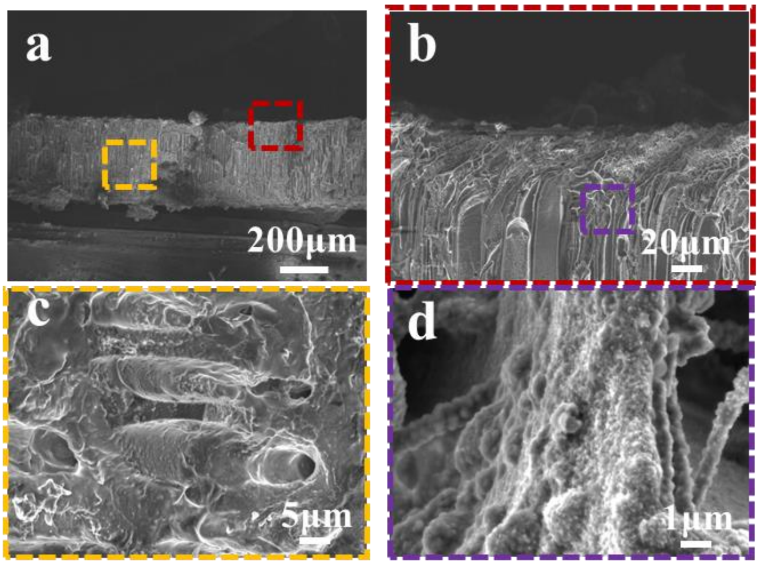

Figure S25. SEM image of Li|VGWs@MCF composite anode after completely Li stripped.

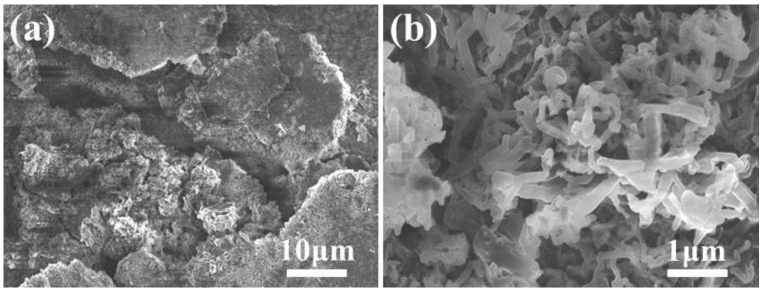

Figure S26. SEM images of Li|Cu composite anode when stripping Li metal beyond 30 mAh cm^{-2}.

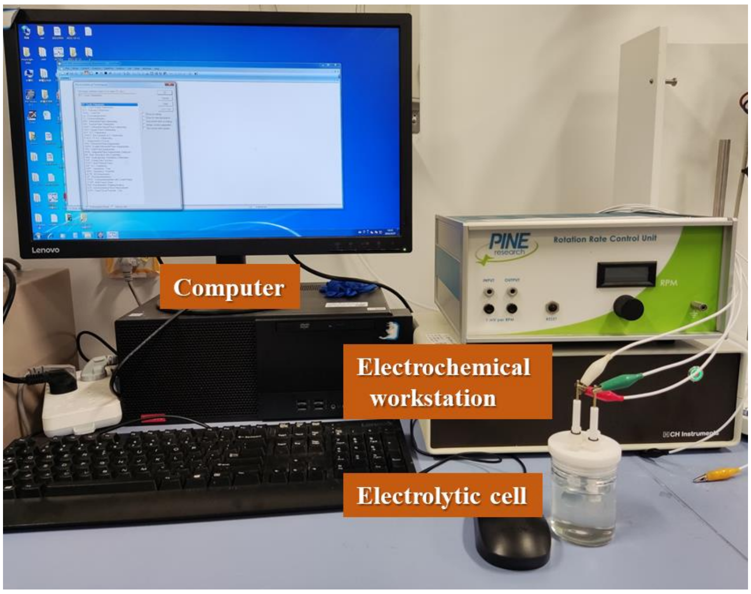

Figure S27. The operando observation equipment conducted in an electrolytic cell.

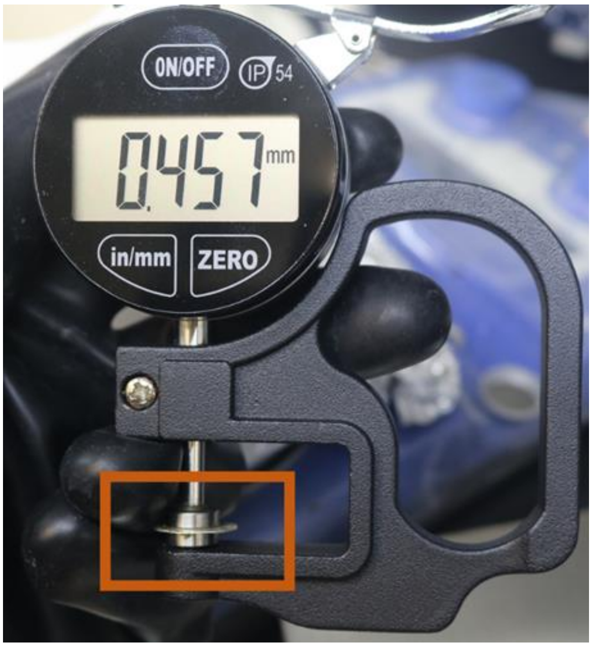

Figure S28. The thickness of the original Li foil.

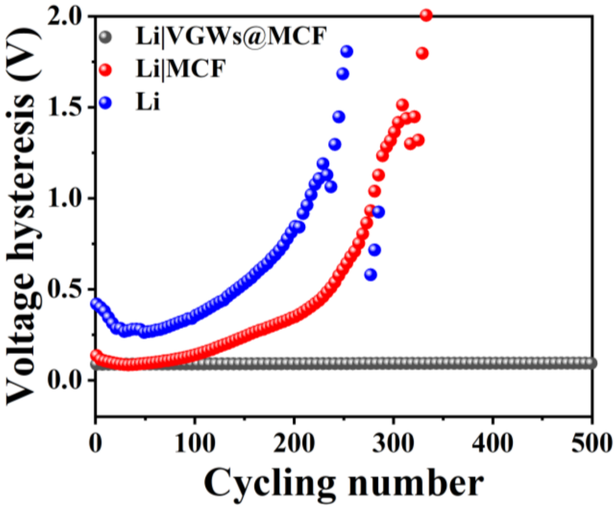

Figure S29. The voltage hysteresis at 1 mA cm^{-2} with the capacity of 1mAh cm^{-2} for bare Li, MCF and VGWs@ MCF symmetrical cells.

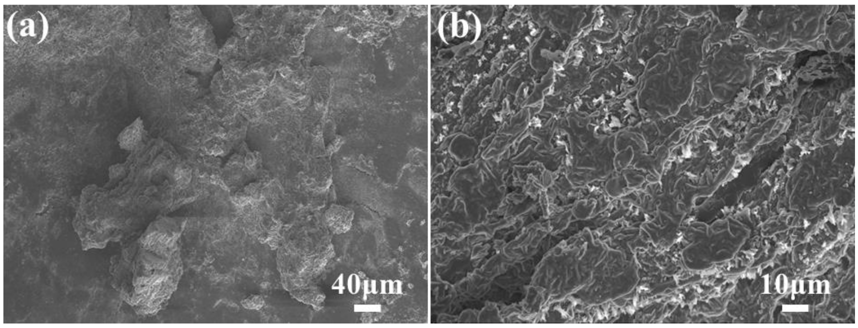

Figure S30. SEM images of bare Li and Li|MCF anode after 250 cycles.

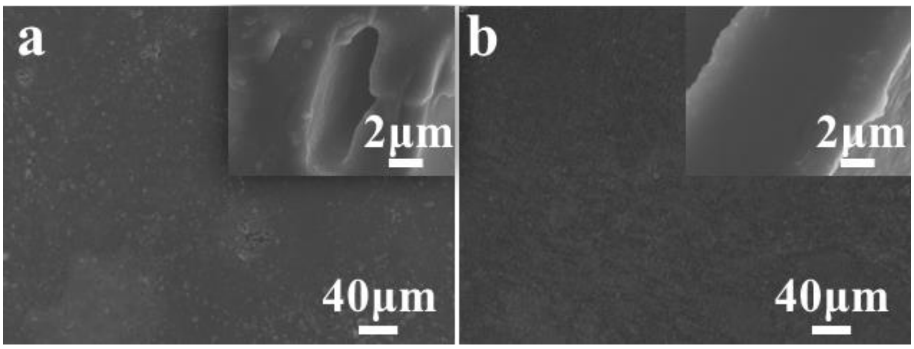

Figure S31. SEM images of Li|VGWs@MCF anode after a) 200 h cycles and b) 500 h cycles.

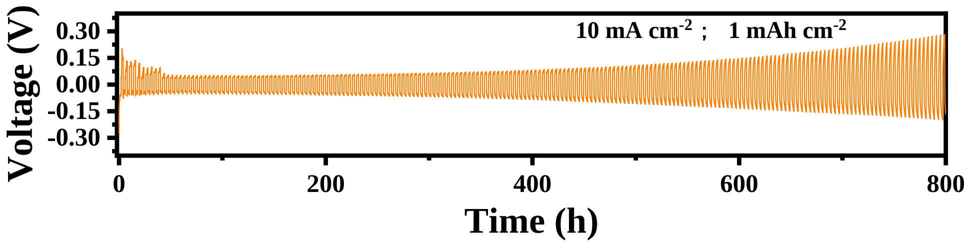

Figure S32. Symmetrical cell of Li|MGWs@MCF anode at 10 mA cm^{-2} with 1 mAh cm^{-2}.

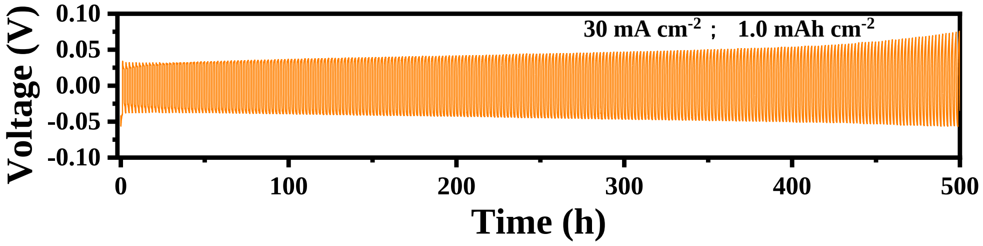

Figure S33. Symmetrical cell of Li|MGWs@MCF anode at 30 mA cm^{-2} with 1 mAh cm^{-2}.

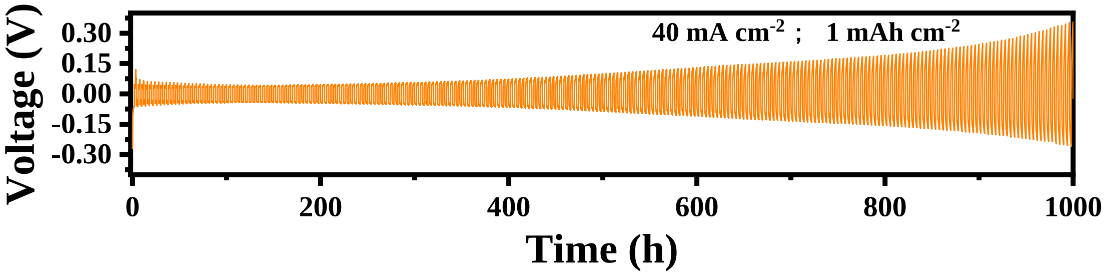

Figure S34. Symmetrical cell of Li|MGWs@MCF anode at 40 mA cm^{-2} with 1 mAh cm^{-2}.

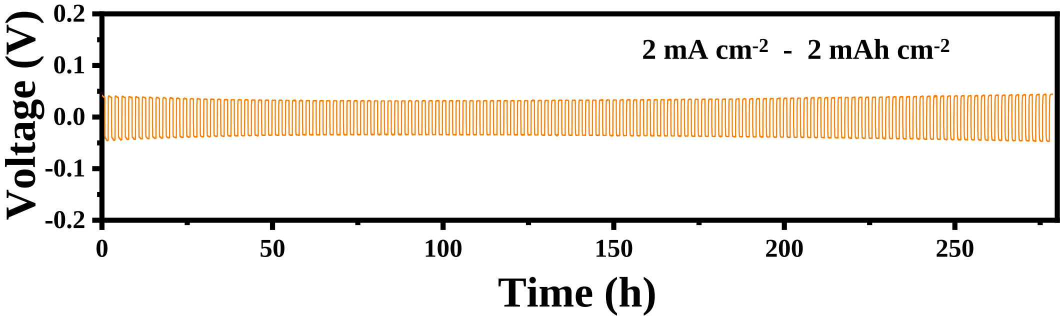

Figure S35. Symmetrical cell of Li|MGWs@MCF anode at 2 mA cm^{-2} with 2 mAh cm^{-2}.

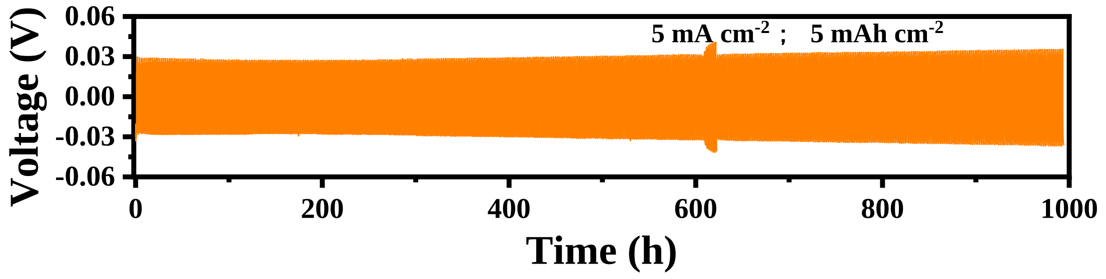

Figure S36. Symmetrical cell of Li|MGWs@MCF anode at 5 mA cm^{-2} with 5 mAh cm^{-2}.

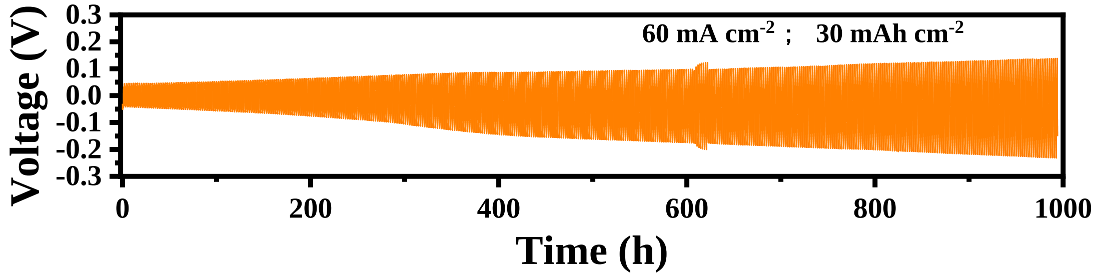

Figure S37. Symmetrical cell of Li|MGWs@MCF anode at 60 mA cm^{-2} with 30 mAh cm^{-2}.

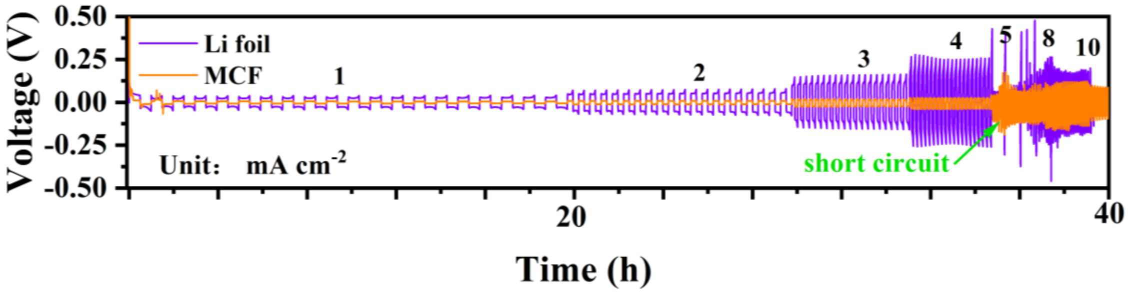

Figure S38. Rate performance of bare Li and Li|MCF anode at various current densities with 1.0 mAh cm^{-2}.

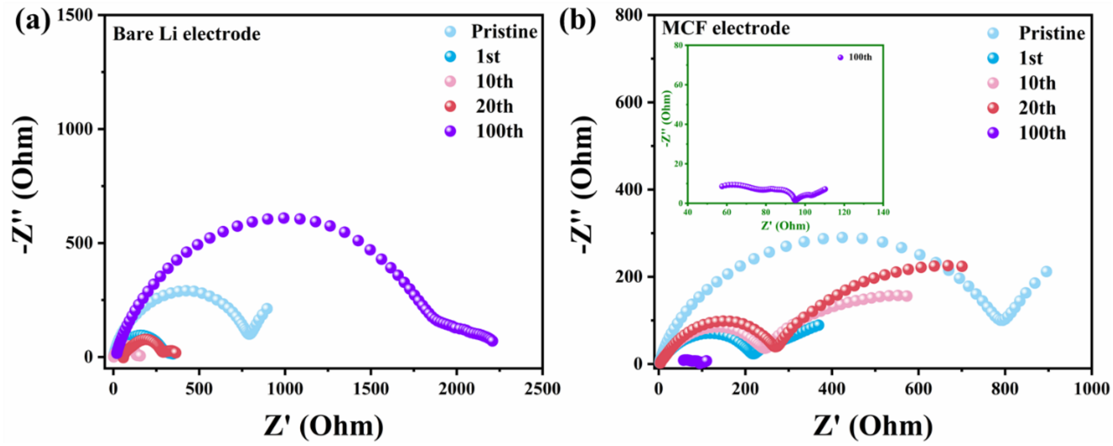

Figure S39. Nyquist plots of symmetrical cells of bare Li and Li|MCF anode at various cycles.

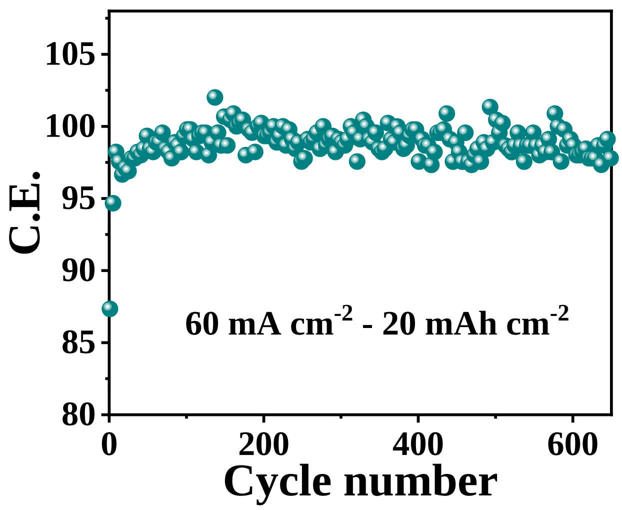

Figure S40. The CE performance of the Li|VGWs@MCF anode at 60 mA cm^{-2} with a capacity of 20 mAh cm^{-2}.

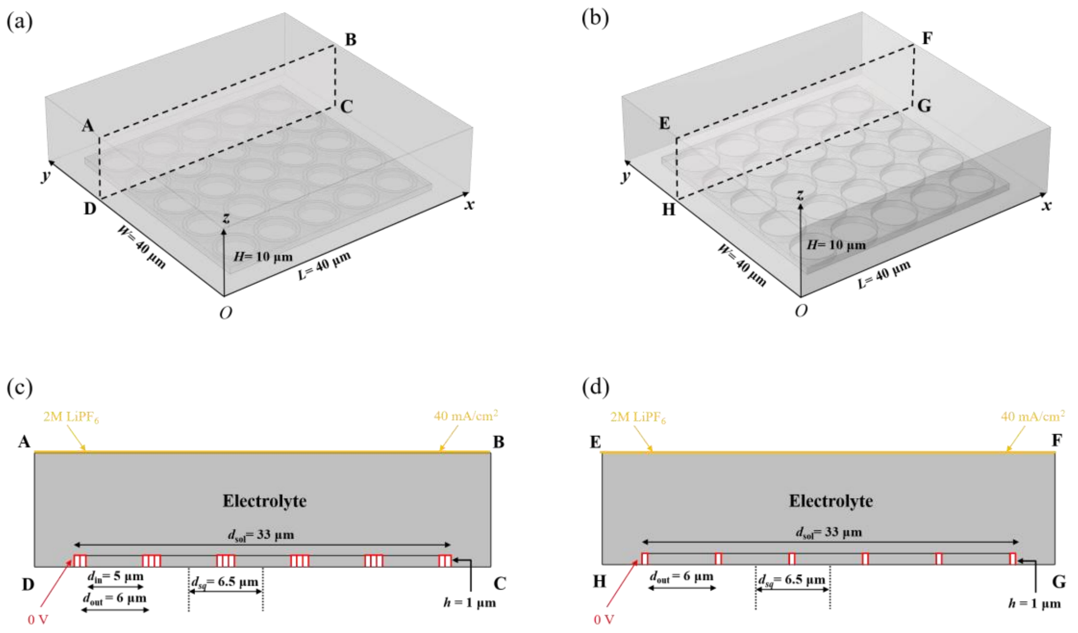

Figure S41. The geometrical structures for (a) VGWs@MCF; (b) MCF. Boundary conditions for (c) VGWs@MCF; (d) MCF. Yellow lines indicate the boundary conditions of initial Li^+ concentration and inflow electric current density. Red lines indicate a boundary electric potential of 0 V.

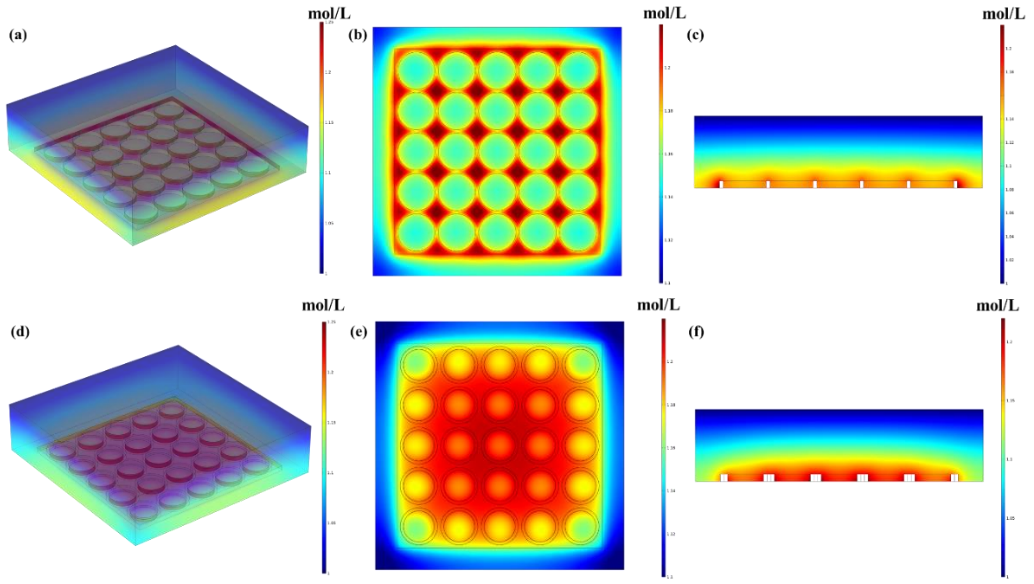

Figure S42. COMSOL multiphysics simulation of Li^+ flux distribution in the (a-c) VGWs@MCF and (d-f) MCF in a 5×5 microchannels.

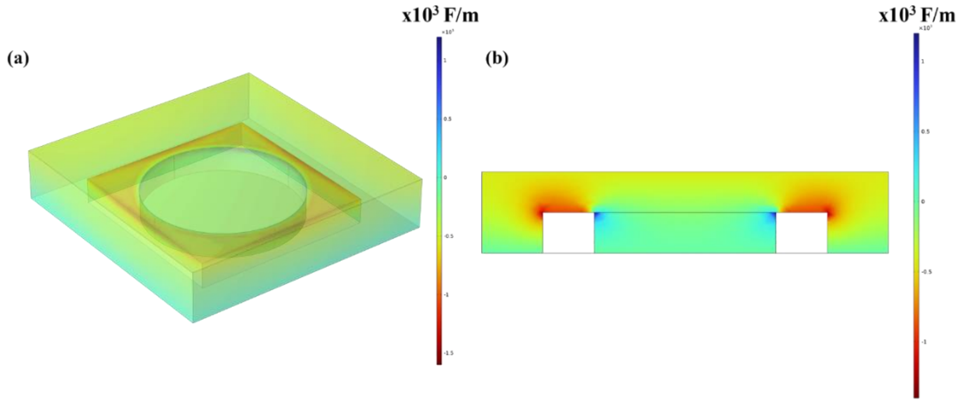

Figure S43. COMSOL Multiphysics simulation of electric field distribution in the MCF (a) 3D profile and (b) cross-section side.

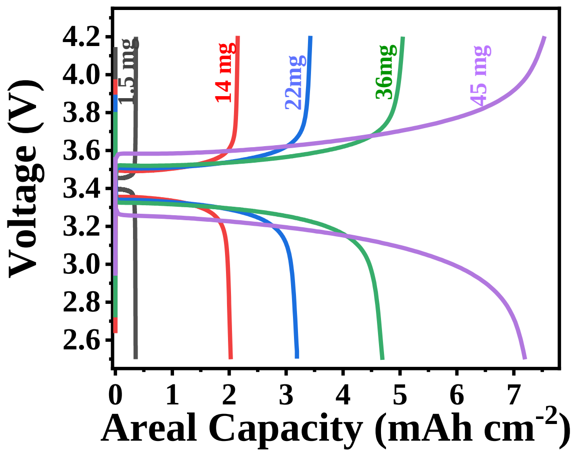

Figure S44. The charge/discharge profiles of the dual vertically aligned electrodes with different LFP loadings and conventional Li|LFP full cell.

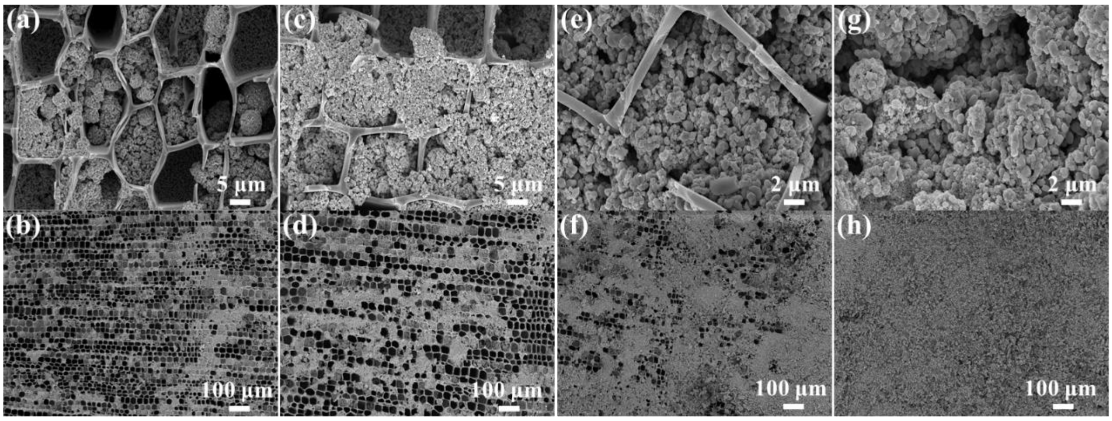

Figure S45. SEM images of LFP|VGWs@MCF electrodes with different loadings. (a, c, e, g) different loadings of LFP for 14, 22, 36, 45 mg cm^{-2}, respectively; and (b, d, f, h) corresponding to the enlarged SEM imges.

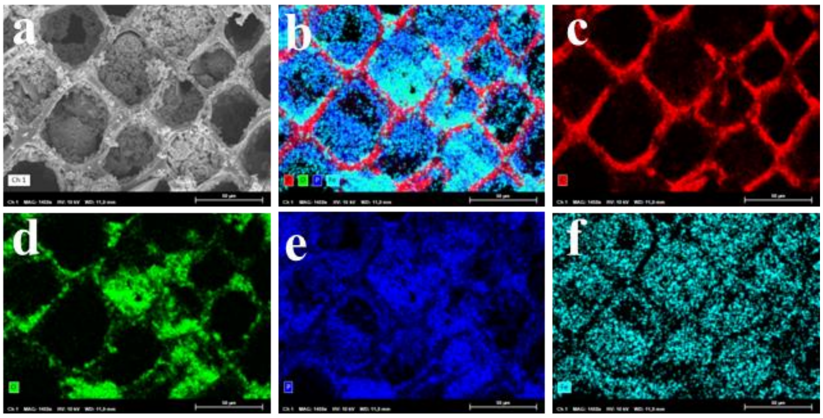

Figure S46. a,) SEM images of LFP/VGWs@MCF cathode, and b-f) the corresponding EDS mapping of C, O, P and Fe.

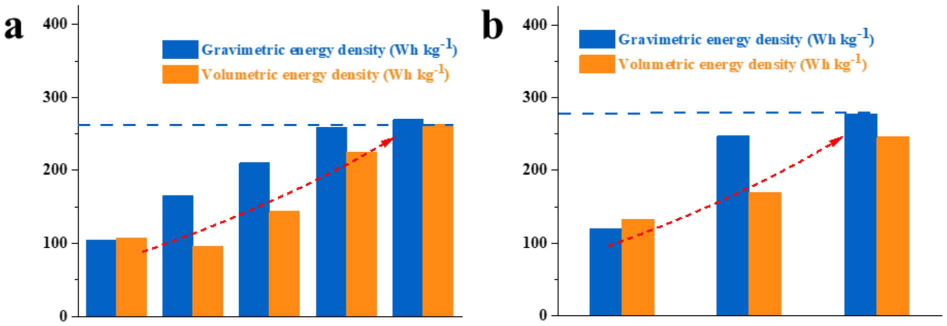

Figure S47. Cell-level energy densities of the a) LFP-Li cell and b) NCM_{811}-Li using a commercial LFP electrode, various LFP/VGWs@MCF electrodes, and NCM/VGWs@MCF electrodes.

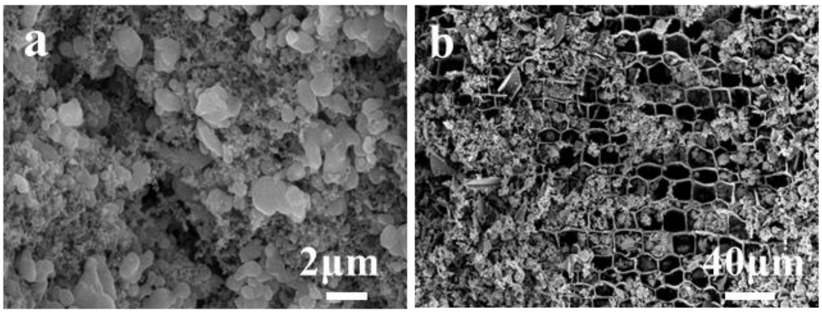

Figure S48. SEM images of LFP/VGWs@MCF cathode after certain cycles.

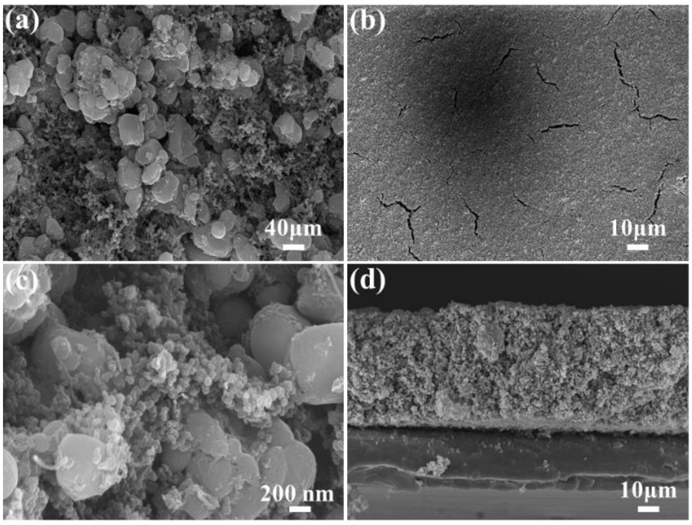

Figure S49. SEM images of LFP electrode from (a, b) top-side and (c, d) cross-section.

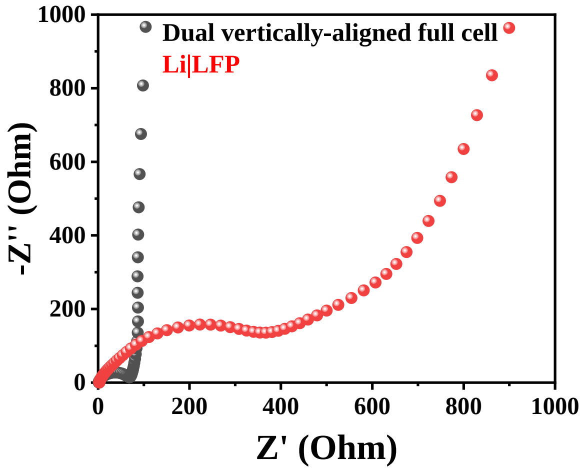

Figure S50. Nyquist plots of the LFP|VGWs@MCF and conventional LFP electrode.

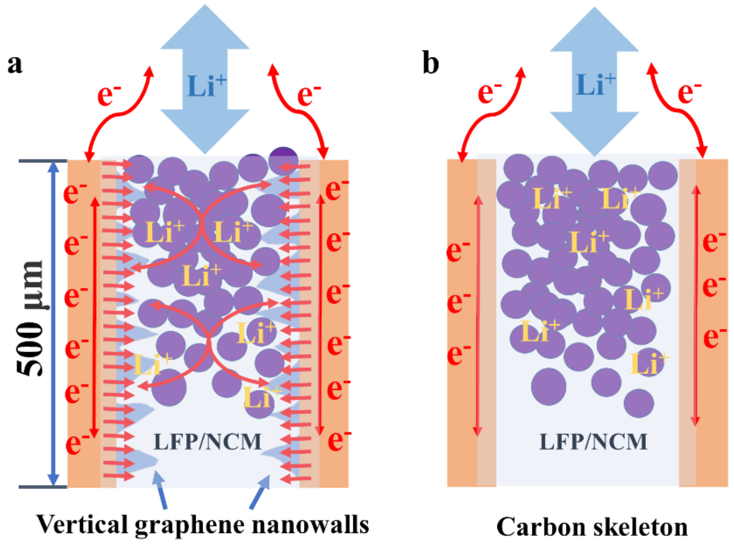

Figure S51. Schematic illustration of a) VGWs@MCF and b) MCF hosts used in LFP/NCM cathode.

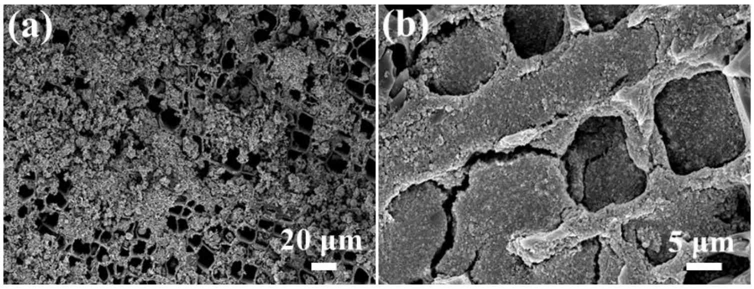

Figure S52. The SEM images of the NMC_{811}|VGWs@MCF with a loading of 35 mg cm^{-2}.

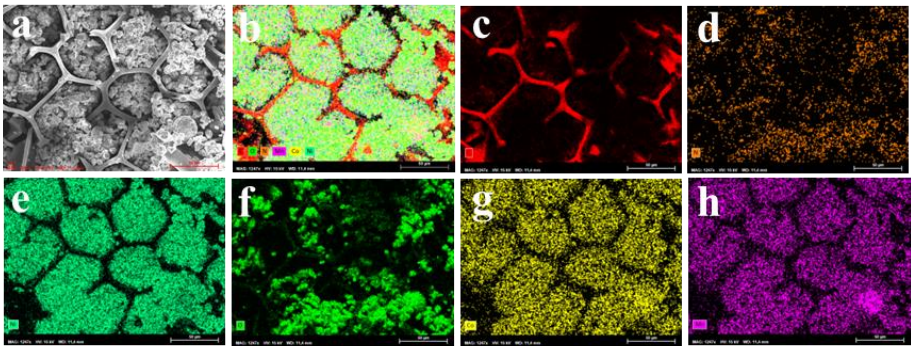

Figure S53. a-h) EDS mapping of NCM/VGWs@MCF cathode.

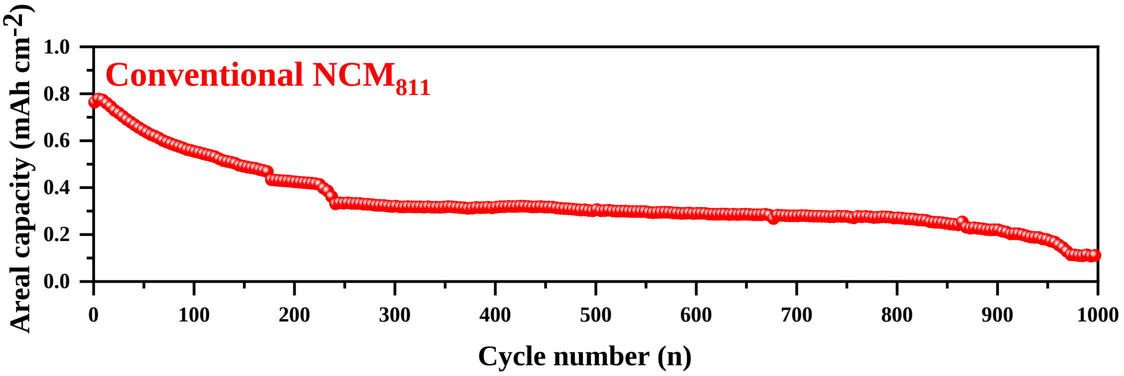

Figure S54. The cycling life of conventional NCM_{811} electrode.

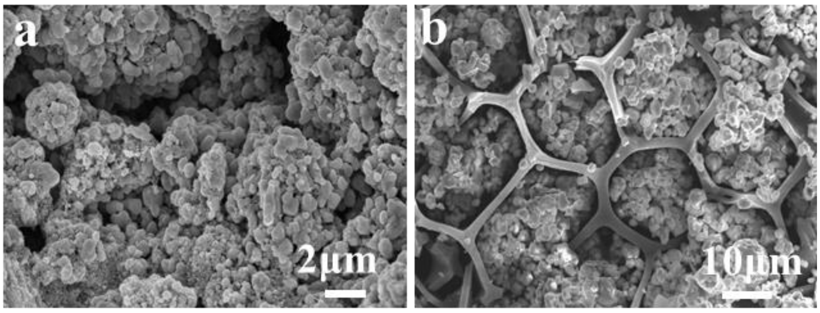

Figure S55. SEM images of NCM_{811}/VGWs@MCF after 200 cycles.

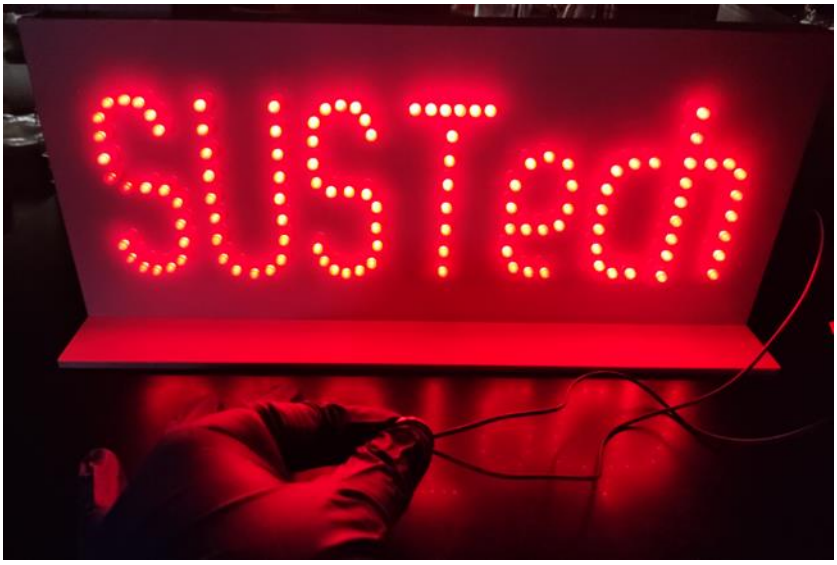

Figure S56. Digital image of an operating LED of SUSTech drived by the high-capacity coin cell.

Table S1 Comparison of electrochemical performances of our Li|MGWs@MCF anode and reported materials for LMAs. (C_d：current density; C: capacity; L:lifespan)

Samples	C_d (mA cm^{-2})	C (mAh cm^{-2})	L (h)	Reference
HPSC	0.5	0.5	2000	Energy Storage Mater. 2022,47, 620-628
VGN/Ni	1	0.5	2000	Adv. Funct. Mater. 2018, 28, 1805638
CTC	10	10	620	Matter, 2022, 5, 1-14
$Ti_3C_2T_x$ MXene@Zn	1	1	300	ACS Nano 2019, 13, 11676-11685
WLC-CNTs	1	2	3100	Nature commun. 2021, 12(1): 1-10.
G-CNF	0.2	0.2	1800	Small 2019, 15, 1903520
$CoSe_2$–NC@CFC	10	20	280	Adv. Sci. 2022, 2104689
N-doped G	5	10	520	Adv. Mater. 2019, 31, 1805334
quicksand-like LMA	20	8	480	ACS Energy Lett. 2021, 6, 3761-3768
SiC/CC	5	5	330	Nano Energy 2022, 94, 106937
CSMF	40	2	100	Adv.Mater. 2021, 33, 2006702
PVA/LRD	30	15	2200	Energy Storage Mater. 2021, 37, 135-142
MgZnO/CNF	50	10	130	Small 2020, 16, 2001992
Al_4Li_9-LiF	20	1	10	Sci. Adv. 2017, 3, e1701301
**MGWs@MCF**	**1**	**1**	**5300**	**This work**
**MGWs@MCF**	**5**	**5**	**1000**	**This work**
**MGWs@MCF**	**10**	**10**	**1000**	**This work**
**MGWs@MCF**	**40**	**1**	**1000**	**This work**
**MGWs@MCF**	**40**	**40**	**520**	**This work**
**MGWs@MCF**	**60**	**30**	**1000**	**This work**

Table S2. The values of R_S, R_{SEI}, and R_{CT} of bare Li, Li|MCF and Li|VGWs@MCF electrode before cycling and cycling.

Samples	R_S (Ω)	R_{SEI}(Ω)	R_{CT}(Ω)
Bare Li, before cycling	2.534	851.5	/
Bare Li, after 1st cycling	3.232	328.1	150.4
Bare Li, after 10th cycling	2.262	235.9	156.3
Bare Li, after 20th cycling	58.35	148.3	67.2
Bare Li, after 100th cycling	20.33	2208	660.7
Li|MCF, before cycling	2.585	502.3	/
Li|MCF, after 1st cycling	3.45	242.5	189.4
Li|MCF, after 10th cycling	3.61	291.2	690.3
Li|MCF, after 20th cycling	4.041	312.6	/
Li|MCF, after 100th cycling	57.58	38.3	19.9
Li|VGWs@MCF, before cycling	19.49	306.4	259.4
LiVGWs@MCF, after 1st cycling	3.291	120.2	164.5
Li|VGWs@MCF, after 10th cycling	4.798	46.4	75.8
Li|VGWs@MCF, after 20th cycling	3.576	15.5	68.5
Li|VGWs@MCF, after 100th cycling	29.77	4.5	11.7

Table S3. Comparison of CE of our Li|VGWs@MCF anode and reported materials for LMAs. (C_d: current density; C: capacity; L: lifespan and CE: coulombic efficiency)

Samples	C_d (mA cm^{-2})	C (mAh cm^{-2})	L (h)	CE (%)	Reference
CoSe@C Aerogel	6	6	100	99.3	Adv. Energy Mater. 2020, 10, 2002654
MgO@GP	15	3.5	100	95.6	Nano Energy 2020, 45,203-209
MgZnO/CNF	1	1	300	97	Small 2020 16, 2001992
Au@ZIF-8	10	1	150	98	Angew. Chem. Int. Ed. 2021, 60, 14040-14050
VA- Ti_3C_2Tx	1	1	500	98.8	Adv. Energy Mater. 2022, 2200072
CCP	12	1	100	91	J. Mater. Chem. A 2019, 7, 13225-13233
q-PET	10	1	50	93	Sci. Adv. 2018, 4, eaar4410
OIFN	2	2	120	96.17	Adv. Mater. 2019, 31, 1806470
CTM	10	1	90	94.2	Adv. Energy Mater. 2019, 9, 1900853
MXene/TiO_2	20	1	500	95.9	J. Mater. Chem. A 2020, 8, 14114-14125
Li_2S/3DCu	4	4	100	98	Adv. Energy Mater. 2020, 10, 1903339
Co@N-G	15	1	150	90.4	Adv. Funct. Mater. 2020, 30, 2000786
GO/ZA	5	3	100	96	Adv. Energy Mater. 2018, 8, 1703152
$Ti_3C_2T_x$ MXene@Zn	1	1	600	97.69	ACS Nano 2019, 13, 11676-11685
G-CNF	5	0.25	500	96	Small 2019, 15, 1903520
**MGWs@MCF**	**1**	**1**	**400**	**99.52**	**This work**
**MGWs@MCF**	**2**	**1**	**800**	**99.4**	**This work**
**MGWs@MCF**	**10**	**1**	**1600**	**99.38**	**This work**
**MGWs@MCF**	**40**	**1**	**1600**	**99.2**	**This work**
**MGWs@MCF**	**60**	**20**	**650**	**98.9**	**This work**

Table S4. Cell components of the commercial LFP electrode and the LFP/ VGWs@MCF electrode. (LFP/ VGWs@MCF :LVM; Current collector: CC)

Component	Commercial electrode		LVM-1 14 mg cm^{-2}		LVM-2 22 mg cm^{-2}		LVM-3 36 mg cm^{-2}		LVM-4 45 mg cm^{-2}	
	Weight (mg cm^{-2})	Thickness (μm)	Weight (mg cm^{-2})	Thickness (μm)	Weight (mg cm^{-2})	Thickness (μm)	Weight (mg cm^{-2})	Thickness (μm)	Weight (mg cm^{-2})	Thickness (μm)
Cu foil/CC	6.52	8	18.56	500	18.56	500	18.56	500	18.56	500
Al foil/CC	4.81	12	/	/	/	/	/	/	/	/
Electrolyte	4.65	/	3.54	/	4.25	/	5.31	/	5.51	/
Separator	1.60	30	1.60	30	1.60	30	1.60	30	1.60	30
Cathode	9.80	62	14.0	/	22.0	/	36.0	/	45.0	/
Anode (Li)	22.0	500	10.6	530	10.6	530	10.6	530	10.6	530
Total Cell	49.38	612	48.3	1060	57.01	1060	72.07	1060	81.27	1060

Table S5. Comparison of areal capacity and rate capability of our LFP-based full cell and previous literature. (R: rate capability, C; C: areal capacity, mAh cm^{-2}; M: mass loading, mg cm^{-2})

Samples	M	R	C	Reference
UCFR-LFP	18	0.5	2.736	Adv. Energy Mater. 2019, 9, 1802930
		1	2.587	
		1.5	2.362	
		2.5	2.084	
		3	1.71	
		3.5	1.357	
MLPE-LFP	8.8	0.2	1.443	Energy Storage Mater. 2019, 19, 88-93
		0.5	1.382	
		1.0	1.311	
		2.0	1.214	
		5.0	1.021	
		10.0	0.352	
PEO@GF/VL-LFP		0.1	1.691	Nano Energy 2019, 61, 567-575
		0.2	1.628	
		0.3	1.554	
		0.5	1.428	
		0.8	1.26	
		1.0	1.05	
		1.5	0.788	
		2.0	0.525	
LFP/CNT/EVA	29.41	0.2	4.85	Adv. Funct. Mater. 2021, 31, 2100434
		0.5	4.73	
		1.0	4.62	
		2.0	4.41	
UCT-LFP	20	0.1	3.132	Energy Storage Mater. 2021, 39, 287-293
		0.5	2.988	
		1.0	2.828	
		2.0	2.664	
		5.0	2.448	
		10	2.178	
Laser-CM-LFP	11.8	0.1	1.82	Chem. Eng. J. 2022, 430, 132810
		0.2	1.82	
		0.5	1.61	
		1	1.40	
		2	1.1	

**LFP|MGWs@MCF**	**45**	**0.1**	**6.61**	**This work**
		**0.2**	**6.38**	
		**0.5**	**6.06**	
		**1**	**5.79**	
		**2**	**5.53**	
		**4**	**5.32**	
		**6**	**4.8**	
		**8**	**4.46**	
		**10**	**4.21**	

Table S6. Cell components of the commercial NCM_{811} electrode and the NCM_{811}/ VGWs@MCF electrode. (NCM_{811}/ VGWs@MCF :NVM; Current collector: CC)

Component	Commercial electrode		NVM-1 20 mg cm^{-2}		NVM-2 35 mg cm^{-2}	
	Weight (mg cm^{-2})	Thickness (μm)	Weight (mg cm^{-2})	Thickness (μm)	Weight (mg cm^{-2})	Thickness (μm)
Cu foil/CC	6.52	8	18.56	500	18.56	500
Al foil/CC	4.81	12	/	/	/	/
Electrolyte	4.65	/	6.32	/	7.85	/
Separator	1.60	30	1.60	30	1.60	30
Cathode	8.9	50	20.0	/	35.0	/
Anode (Li)	22.0	457	10.6	530	10.6	530
Total Cell	48.48	557	57.08	1060	73.61	1060

Table S7 Comparison of areal capacity and rate capability of our NCM_{811}-based full cell and previous literature. (R: rate capability, C; C: areal capacity, mAh cm^{-2}; M: mass loading, mg cm^{-2}; L: cycling lifespan, n)

Samples	M	R	C	L	Reference
3DP-NCM_{811}	36.6	0.1	7.48	30	Energy & Fuels, 2020.
$LiBO_2$@Li/NCM_{811}	9.9	0.5	1.36	300	Small 2022, 18, 2106427
PPS@Li/NCM_{811}	25.4	0.2	4.2	200	Adv. Energy Mater. 2019, 9, 1900704
PCNM@ Li/NCM_{811}	20	0.2	3.75	120	Nano Research, 2021,14, 3585-3597
LiDFOB/NCM_{811}	30	0.33	6	50	Nano Energy, 2022, 96,107122
Li/PVDF-PAN/ NCM_{811}	20.9	0.1	4	150	Nano Lett. 2021, 21, 4757-4764
SCCE Electrolyte/ NCM_{811}	16.7	0.3	3. 4	200	Adv. Mater. 2020, 32, 2001740
FDMA@Li/ NCM_{811}	17.95	0.25	3. 5	500	Nature commun. 2020.11, 1-11
Self-smoothing Li@C/ NCM_{811}	19.23	0.33	4	200	Nature nanotech. 2019, 14, 594-601
**Dual vertically aligned Li|NCM_{811}**	**35**	**1.0**	**3**	**420**	**This work**
	**20**	**1.0**	**2**	**420**	**This work**

Refeerence:

[1] J. Wu, Z. Ju, X. Zhang, K. J. Takeuchi, A. C. Marschilok, E. S. Takeuchi, and G. Yu. Building Efficient Ion Pathway in Highly Densified Thick Electrodes with High Gravimetric and Volumetric Energy Densities. Nano Letters 2021, 21, 9339-9346.
